# Systemic dysregulation and molecular insights into poor influenza vaccine response in the aging population

**DOI:** 10.1126/sciadv.adq7006

**Published:** 2024-09-27

**Authors:** Saumya Kumar, Martijn Zoodsma, Nhan Nguyen, Rodrigo Pedroso, Stephanie Trittel, Peggy Riese, Javier Botey-Bataller, Liang Zhou, Ahmed Alaswad, Haroon Arshad, Mihai G. Netea, Cheng-Jian Xu, Frank Pessler, Carlos A. Guzmán, Luis Graca, Yang Li

**Affiliations:** ^1^ Centre for Individualised Infection Medicine (CiiM), a joint venture between the Helmholtz Centre for Infection Research (HZI) and Hannover Medical School (MHH), Hannover, Germany.; ^2^ TWINCORE, a joint venture between the Helmholtz-Centre for Infection Research (HZI) and the Hannover Medical School (MHH), Hannover, Germany.; ^3^ Instituto de Medicina Molecular João Lobo Antunes, Faculdade de Medicina, Universidade de Lisboa, Lisboa, Portugal.; ^4^ Department of Vaccinology and Applied Microbiology, Helmholtz Centre for Infection Research, Braunschweig, Germany.; ^5^ Department of Internal Medicine and Radboud Center for Infectious Diseases, Radboud University Medical Center, Nijmegen, Netherlands.; ^6^ Department of Immunology and Metabolism, Life and Medical Sciences Institute (LIMES), University of Bonn, Bonn, Germany.; ^7^ Research Group Biomarkers for Infectious Diseases, TWINCORE, Hannover, Germany.; ^8^ Cluster of Excellence Resolving Infection Susceptibility (RESIST; EXC 2155), Hannover Medical School, Hannover, Germany.; ^9^ Lower Saxony Center for Artificial Intelligence and Causal Methods in Medicine (CAIMed), Hannover, Germany.

## Abstract

Vaccination-induced protection against influenza is greatly diminished and increasingly heterogeneous with age. We investigated longitudinally (up to five time points) a cohort of 234 vaccinated >65-year-old vaccinees with adjuvanted vaccine FluAd across two independent seasons. System-level analyses of multiomics datasets measuring six modalities and serological data revealed that poor responders lacked time-dependent changes in response to vaccination as observed in responders, suggestive of systemic dysregulation in poor responders. Multiomics integration revealed key molecules and their likely role in vaccination response. High prevaccination plasma interleukin-15 (IL-15) concentrations negatively associated with antibody production, further supported by experimental validation in mice revealing an IL-15–driven natural killer cell axis explaining the suppressive role in vaccine-induced antibody production as observed in poor responders. We propose a subset of long-chain fatty acids as modulators of persistent inflammation in poor responders. Our findings provide a potential link between low-grade chronic inflammation and poor vaccination response and open avenues for possible pharmacological interventions to enhance vaccine responses.

## Teaser

Systemic dysregulation and key molecules in elderly flu vaccine response highlight intervention targets.

## INTRODUCTION

Seasonal influenza vaccination is currently the most effective approach for protection against infection, particularly in the aging population (*1*–*3*). Despite vaccination, host heterogeneity in protection has been observed (*3*, *4*). The reduced efficiency in generating protective immunity in the elderly has been attributed to the aging immune system, which is often characterized by persistent low-grade inflammation and immunosenescence (*5*). Comparisons among aging population and young have revealed higher proportions of inflammatory monocytes and cytotoxic natural killer (NK) cells in the aging population compared to the younger population, along with lower B cell responses attributed to intrinsic defects in T and B cells (*6*, *7*). In addition, the elderly accumulate proinflammatory B cells that fail to effectively respond to influenza vaccination (*8*). At the molecular level, lower activating protein 1 (AP1) transcription factors activity coupled with enhanced antiviral and interferon signaling in myeloid cells has been linked with the AS03-adjuvated H5N1 pandemic vaccine response (*9*). However, in the younger individuals (<50 years), a prevaccination high inflammatory status driven by nuclear factor κB signaling has been associated with a stronger antibody response (*10*). Differential metabolic regulation of the immune response to vaccination has also been observed between aging population and younger population, with fatty acids and amino acids playing roles in the vaccine response (*11*, *12*). After vaccination, younger high responders (HRs) showed reduced levels of polyunsaturated fatty acids, while cholesteryl esters accumulated more in elderly HRs. In addition, purine metabolism and glycine, serine, and threonine metabolism have also been associated with vaccine response outcomes (*11*). These studies indicate a complex and age-dependent differential regulation of the antibody response to vaccination in the aging population. However, a systematic multiomics examination of host response differences within the aging population leading to differential vaccine response has largely remained unexplored.

Here, we used a systems approach to examine multiple omics modalities in relation to the differential responsiveness to the FluAd vaccine, a trivalent inactivated adjuvanted influenza vaccine in a cohort of 234 donors aged >65 years, spanning two independent influenza seasons (*7*, *13*–*15*) (with no overlaps between donors from the two seasons). Using pre- and postvaccination serological data, donors were categorized as HRs (>4-fold rise) and low responders (LRs) (<4-fold rise) of each strain and also as triple responders (TRs) (>4-fold rise in antibody titers against all three strains) and total nonresponders (NRs) (<4-fold rise in antibody titers against all three strains). We included longitudinal samples from each donor across two different influenza seasons to identify the canonical molecules signifying robust host response differences to vaccination. We show distinct changes in the plasma proteome and metabolome abundance upon vaccination in HRs and LRs as well as total triple and total NRs. The transcriptional signatures of the triple and total NRs showed a chronic inflammatory signature in total NRs. Furthermore, we identified prevaccination protein and metabolite biomarkers associated with poor vaccine response describing a link between chronic inflammation and reduced antibody production as observed in poor responders. Results from this study provide insight into the immunological processes driving differential responsiveness to vaccination and will open opportunities for improved vaccine design as well as modulation of the immune system to enhance the vaccine response in the aging population.

## RESULTS

### Age and gender partially explain variation in vaccine response within the elderly population

**Fig. 1.**
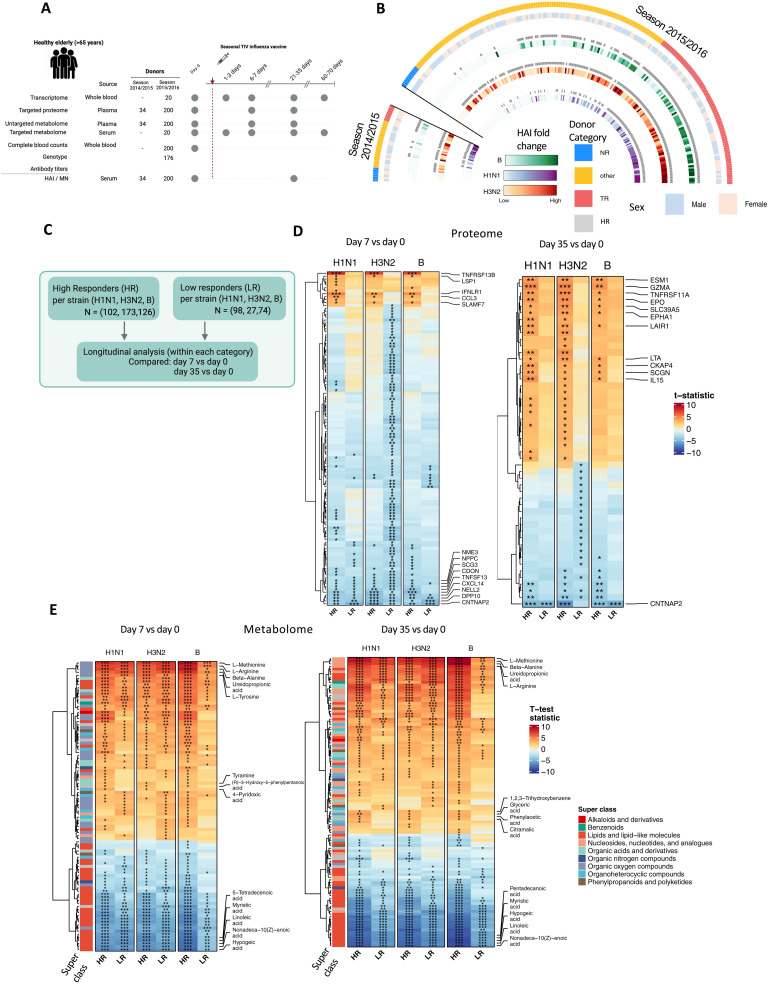
Influenza vaccination cohort overview and vaccine response changes in HRs and LRs of each strain. (**A**) Overview of the different omics datasets generated in this study. We generated multimodal datasets covering the transcriptome, proteome, and metabolome response to influenza vaccination from up to 234 individuals for different time points spanning both before and after vaccination, covering two independent seasons of influenza vaccination. The serological data are adapted from earlier publications on the same cohort. (**B**) Circos plot showing the serological response to TIV. Each heatmap depicts antibody fold change (HAI titers) against the three strains included in the vaccine (H3N2, H1N1, and B). Donors were classified as HR for each strain (gray tiles), LRs (lack of gray tiles), TR, NR, or Other on the basis of their serological response (outer-colored tiles). (**C**) Schematic overview of the analysis. Schematics created using Biorender.com. (**D**) Heatmap showing longitudinal differential protein abundance (left, day 7 versus day 0; right, day 35 versus day 0) in HRs and LRs of each strain. HRs for H1N1 (*N* = 102), LRs for H1N1 (*N* = 98); HRs for H3N2 (*N* = 173), LRs for H3N2 (*N* = 27); HRs for B strain (*N* = 126), LRs for B strain (*N* = 74). Proteins labeled are significantly differentially abundant in either HRs or both (HRs and LRs) of each strain in each longitudinal comparison and common across all three strains. (**E**) Heatmap showing longitudinal differential metabolite abundance, same as (D) with superclass annotations per metabolite as annotated by the Human Metabolome Database (HMDB). (*P_adj_ < 0.05, **P_adj_ < 0.01, and ***P_adj_ < 0.001.)

We collected whole blood and plasma samples from 234 trivalent inactivated influenza vaccine (TIV) vaccinees aged >65 across two influenza seasons and generated multimodal datasets covering whole blood transcriptome, plasma proteome, plasma/serum metabolome, and serology from early, acute, and up to 2 months after vaccination, was characterized (Fig. 1A and fig. S1A). Hemagglutination inhibition (HAI) titers and microneutralization (MN) titers were measured against each strain (H1N1, H3N2, and B) included in the vaccine (*7*) (see Materials and Methods). On the basis of the fold change in the measured serological data, donors were categorized as either HRs or LRs (>4-fold rise in antibody tires against each strain) and also as TRs (>4-fold rise in antibody titers against all three strains), NRs (<4-fold rise in titers against all three strains), or Other (>4-fold rise in titers against one or two of the vaccine strains (Fig. 1B). We used the larger cohort (season 2015/2016, *n* = 200) as a discovery cohort and the smaller cohort to replicate our findings (season 2014/2015, *n* = 34). Donors from both cohorts did not overlap, and longitudinal samples from each donor were collected.

We observed an additional layer of heterogeneity among the donors based on prevaccination antibody titers. Previous studies have highlighted the impact of high prevaccination HAI titers that may lead to a reduced antibody fold change upon vaccination (*6*, *16*–*18*). In our cohort, approximately one-third of the donors (H1N1: 36%, B: 34%, and H3N2: 33%) exhibited this pattern. However, donors across all the three categories (TRs and others) still showed a high antibody response to vaccination independent of their prevaccination titers. This suggests that prevaccination titers are not the sole factor influencing the vaccine response (Fig. 1B). We also observe positive correlations among antibody titers against the three influenza strains and positive correlations between HAI titers and MN titers for each strain (fig. S1, B and C). No statistically significant differences in serological responses between males and females were found (fig. S1D). Across two seasons, we observed a statistically significant negative correlation of antibody titers against the H1N1 strain with age of participants but not for the other two strains (fig. S1E). These observed patterns were replicated in the smaller cohort.

### Postvaccination changes in plasma molecules highlight key proteins and metabolites differing in HRs and LRs

We first evaluated the differences in up to 311 circulating proteins measured using OLINK Explore Inflammation panel in 200 donors categorized as HRs and LRs against each strain (Fig. 1C). Prevaccination proteomes between HRs and LRs did not result in statistically significant differentially abundant proteins. However, statistically significant differences were found when examining longitudinal changes (day 7 versus day 0 and day 35 versus day 0) in HRs and LRs separately (Fig. 1D). In day 7 to day 0 comparison, HRs against each strain showed up-regulation of tumor necrosis factor receptor superfamily member 13B (TNFRSF13B), C-C motif chemokine 3 (CCL3), Interferon lambda receptor 1 (IFNLR1), and SLAM family member 7 (SLAMF7) (moderated *t* test, *P*_adj_ <0.05), while these did not reach statistical significance in LRs. TNFRSF13B (also known as TACI) has been widely described for its role in B cell proliferation and plasma cell differentiation (*19*, *20*). The role of increased IFNLR1 expression in human alveolar macrophages and monocyte-derived macrophages in inhibiting influenza infection has also been described (*21*, *22*). In particular, IFNLR1 is down-regulated in LRs across all three strains (Fig. 1D and table S1). Comparing day 35 versus day 0, we observed higher number of statistically significantly up-regulated proteins in HRs (Fig. 1D). These proteins were also up-regulated in LRs; however, the changes were not statistically significant. To identify the persistent changes in response to vaccination in HRs, we examined the shared changes in protein abundance between day 7 versus day 0 and day 35 versus day 0 (fig. S2A).

We also assessed untargeted metabolomic data measured by liquid chromatography–mass spectrometry (LC-MS) and focused specifically on 192 endogenous metabolites (see Materials and Methods). Examining differential abundance of prevaccination metabolites in HRs and LRs did not show statistically significant differentially abundant metabolites. However, evaluating longitudinal differences separately in HRs and LRs, we found many statistically significant differentially abundant metabolites after vaccination. We found consistent up-regulation of certain amino acids (methionine, arginine, and tyrosine) and down-regulation of fatty acyls in both HRs and LRs (comparison day 7 or day 35 versus day 0) evident from enrichment analysis (Fig. 1E and table S2). In addition, 4-pyridoxic acid, a metabolite of vitamin B6, is up-regulated only in HRs for all three strains in day 7 versus day 0 comparison. The role of vitamin B6 in antibody production and T cell immunity has been previously described (*23*). Tyramine, a metabolite of tyrosine, has also been described for its immunomodulatory roles (*24*) and is up-regulated in HRs (Fig. 1E).

These observations from proteome and metabolome highlight key molecules involved in the vaccination response (as seen in HRs) with their previously described role in immune response and function. In addition, these molecules either are missed or appear insufficient to mount a protective immune response in LRs at both day 7 and day 35. This suggests an impairment of the immune response to vaccination in LRs.

### Plasma molecular changes in TRs highlight important proteins and metabolites shaping the immune response to vaccination

**Fig. 2.**
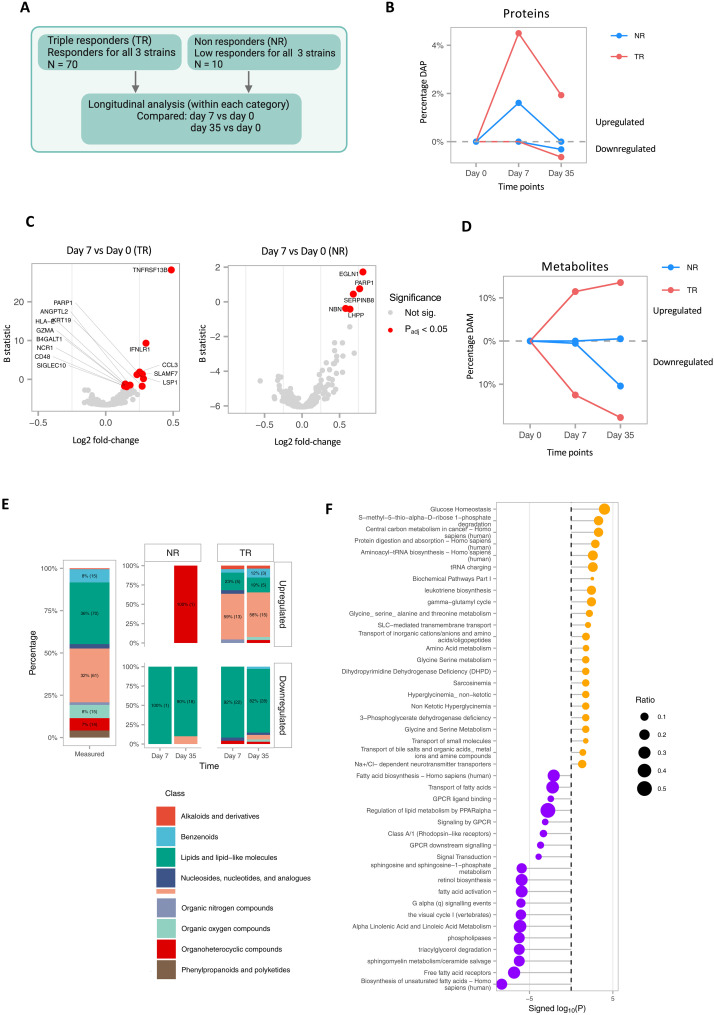
Plasma proteins and metabolite changes upon vaccination distinct in TRs and NRs. (**A**) Schematic overview of the analysis. Schematic created using Biorender.com. (**B**) Line plot showing significant changes (*P*_adj_ < 0.05) in protein abundance in TRs (*N* = 71) and NRs (*N* = 10) after vaccination. TRs show a higher percentage of significantly up-regulated proteins, while NRs show a comparably dampened response. (DAPs: Differentially Abundant Proteins). (**C**) Volcano plot of proteins up-regulated in TRs and NRs 7 days after vaccination with significant proteins colored in red and labeled. Proteins were considered significant at *P*_adj_ < 0.05. (**D**) Line plot showing significant changes in metabolite abundance in TRs (*N* = 71) and NRs (*N* = 10) after vaccination. DAMs, differentially abundant metabolites. (**E**) Significantly differentially regulated metabolites categorized on the basis of their class as annotated by the HMDB. The “Measured” column represents the total universe of metabolites that were measured. The TR and NR columns indicate significantly up or down regulated metabolites at each time point compared to day 0. (**F**) Pathways enriched for metabolites with increased or decreased abundance 7 days after vaccination in TRs. Enrichment of pathways was calculated using IMPALA. Pathways with adjusted *P* values <0.05 are shown.

Having accessed vaccination response in HRs and LRs against individual strains (Fig. 1, D and E), we next evaluated the differential abundance of proteins and metabolites based on donors classified as TRs (responders to all three strains, *N* = 71) and NRs (nonresponders to all three strains, *N* = 10) (Fig. 2A). Comparing TRs to NRs, we found higher CLEC7A in TRs in the discovery cohort (moderated *t* test, adj. *P* = 0.03) but not in the replication cohort possibly because of the small effect size (fig. S2B, top). We next evaluated longitudinal or vaccine response changes in proteome and metabolome in TRs and NRs separately (Fig. 2A). Upon separate examination of protein time dynamics in TRs and NRs, changes in protein abundance in both TRs and NRs 7 days after vaccination were observed (Fig. 2B and table S3). In TRs, 14 proteins were up-regulated at 7 days after vaccination (Fig. 2C), including the proteins identified previously in HRs defined by antibody responses for each strain (Fig. 1D); three of these proteins also showed increased abundance in the replication cohort (fig. S2B, bottom). Up-regulation of additional molecules e.g., CD48, Sialic acid-binding Ig-like lectin 10 (SIGLEC10), Granzyme A (GZMA), and HLA class I histocompatibility antigen, alpha chain E (HLA-E), and their described role in activation and anti-inflammation (*25*–*28*) may indicate optimum shaping of the immune response to vaccination in TRs, while these proteins did not change in NRs upon vaccination. We therefore examined the prevaccination abundance of these proteins in NRs and TRs but did not find statistically significant differences (fig. S2C). Instead, five distinct proteins were up-regulated in NRs at day 7 after vaccination, including poly(adenosine diphosphate–ribose) polymerase 1 (PARP1), Egl nine homolog 1 (EGLN1), and Serpin B8 (SERPINB8) (Fig. 2C). PARP1 plays a role in oxidative stress-induced inflammation and in recruiting NK cells in a viral response (*29*, *30*). EGLN1/PHD2 and SERPINB8 are described for their role in hypoxia and platelet function (*31*–*34*) anti-inflammatory roles. At day 35, we found relatively fewer differentially abundant proteins (fig. S2D). These results highlight additional proteins along with the proteins identified in HRs against each strain as important for complete protection against the three strains, as observed in TRs.

Upon examination of metabolites, no statistically significant differentially abundant metabolites between TRs and NRs were found, irrespective of the time points. However, longitudinal abundance changes in metabolites in TRs at days 7 and 35 compared to prevaccination were observed, whereas this was less evident in NRs (Fig. 2D and table S4). Taxonomic classification of these metabolites showed statistically significant increase in the abundance of organic acids, particularly amino acids, crucial for immune function both at 7 and 35 days after vaccination in TRs. (*35*). Metabolites with decreased abundance at 7 and 35 days after vaccination were lipids and lipid-like molecules, specifically fatty acyls (Fig. 2E and fig. S3). These findings were validated in the replication cohort (fig. S3A). We found that four of five up-regulated lipids and lipid-like molecules in TRs are bile acids, previously described for their role in influenza vaccination (*36*). NRs did not show any statistically significant differences in metabolite abundance 7 days after vaccination but showed a decrease in lipid abundance 35 days after vaccination (Fig. 2, D and E). Pathway over-representation analysis in TRs at day 7 showed statistically significant up-regulation of metabolic pathways involving amino acid, glycine/serine metabolism, and glucose homeostasis (Fig. 2F and table S5), and down-regulated metabolic pathways were related to fatty acids, linoleic acid metabolism, and signal transduction, among others. These results show that total NRs lacked the increased and sustained abundance of amino acids, unlike LRs of individual strains, suggesting a metabolic dysregulation in total NRs upon vaccination.

### Persistence inflammatory transcriptome signatures describe differences in NRs and TRs

**Fig. 3.**
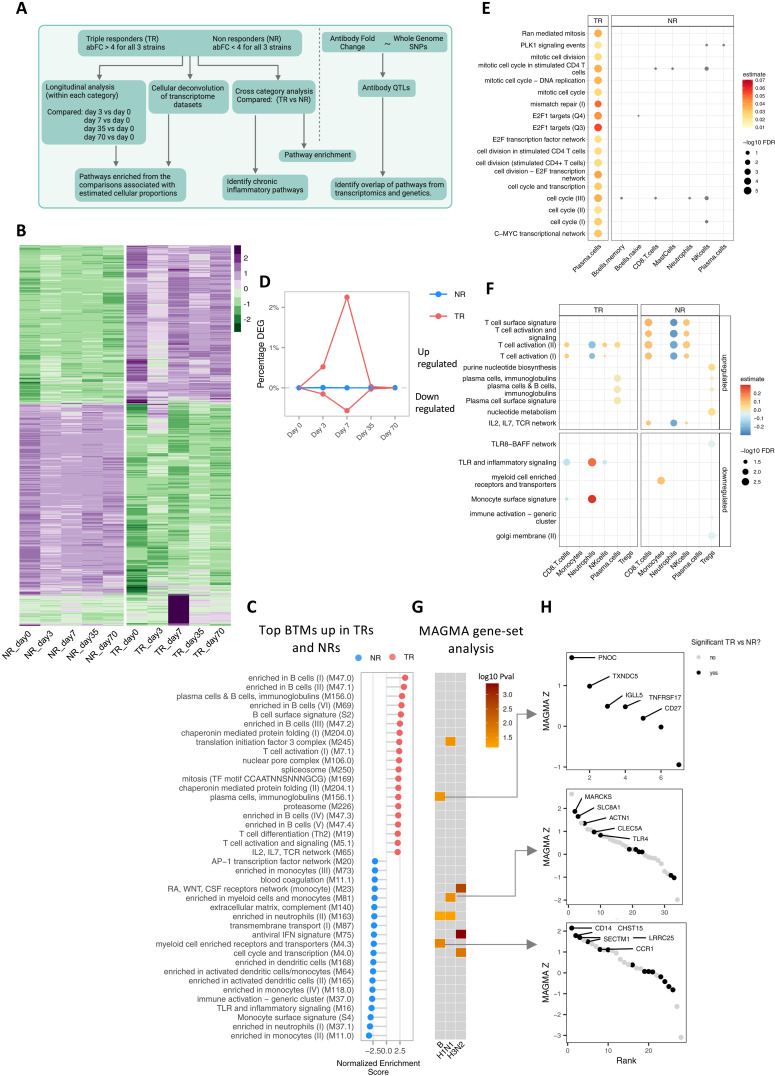
Pathway enrichment time and transcriptomics vaccine response differences in highest and lowest responders. (**A**) Schematic overview of the analysis. Schematic created using Biorender.com. (**B**) Heatmap showing mean gene expression across 10 TRs and 10 NRs for each time point. Each row is a gene significantly differentially expressed between TRs and NRs, purple indicating higher gene expression, green indicating lower gene expression (*P*_adj_ < 0.01). (**C**) Top 40 BTMs based on GSEA in TRs versus NRs. Positive (red) normalized enrichment scores (NES) correspond to BTMs up-regulated in TRs; negative NES corresponds to BTMs up-regulated in NRs. (**D**) Line plot summarizing changing gene expression in TRs (red) and NRs (blue) upon vaccination indicated by the percentage of significantly DEGs over time compared to prevaccination. (**E**) Association of deconvoluted cell proportions with cell cycle BTMs and (**F**) cell type signature BTMs at 7 days after vaccination. Size of the dots represent adjusted *P* values from linear mixed models, while color represents the estimate from the model. Associations with *P*_adj_ < 0.05 plotted for TRs, while all associations for NRs with cell cycle BTMs in gray (not significant) in (E). (**G**) MAGMA gene-set level scoring of the BTMs listed in (C). The genetic basis of the serological response to vaccination was assessed. MAGMA was used to collapse variant-level *P* values to suggestive gene-set-level *P* values (nominal *P* < 0.1). Shown in colors are the log_10_(*P*) values of the suggestive pathways; gray colors represent nonsignificance. (**H**) Gene rankings based on MAGMA’s gene-level *z*-score (Materials and Methods) for three selected pathways that show suggestive genetic regulation. Genes were ranked (*x* axis) on their *z*-score (*y* axis) from MAGMA. The color is based on whether the gene is also significantly differentially expressed between TRs and NRs (black) or not (gray) (*P*_adj_ < 0.05).

After accessing the plasma differences in TRs and NRs (Fig. 2), we examined the transcriptome of a subset of TRs and NRs to characterize functional differences from the same cohort (Fig. 3A). Differential gene expression analysis between the groups and the transcriptomic dynamics of vaccine response resulted in 1466 statistically significant differentially expressed genes (DEGs) (*P*_adj_ < 0.01, Fig. 3B). Gene set enrichment analysis (GSEA) using blood transcriptome modules (BTMs) (*37*) showed positively enriched modules related to T and B cells and T cell activation in TRs, whereas NRs up-regulated inflammatory and inflammatory signaling modules (Fig. 3C and table S6). These pathways were consistently up-regulated in TRs and NRs at all time points, including prevaccination. Deconvolution of transcriptome data in immune cell proportions for each donor also reflected these differences, whereas TRs had higher proportions of T and B cells, NRs showed elevated proportions of neutrophils [CIBERSORT (*38*); Figure S4A and S4B). These results agree with the observed trend of higher neutrophils in NRs at the prevaccination time point based on complete blood counts for all 200 donors (fig. S4C). Despite the enrichment of monocyte-related BTMs in NRs, we did not observe differences in monocyte proportions between TRs and NRs in deconvolution or cell blood counts. This suggests higher activity of monocytes in NRs irrespective of cell proportions (Fig. 3C and fig. S4, A and C). In addition, evaluating only prevaccination BTMs that separate TRs from NRs, we found TRs enriched for T cell activation and signaling, T helper 2 differentiation and plasma/B cell modules (fig. S4A and table S7). This agrees with the previous report stating that prevaccination inflammatory BTMs do not characterize aging population responders (*10*, *39*). Instead, we observed that TRs were less enriched for inflammatory modules compared to NRs (fig. S5, A and B). In summary, prevaccination BTMs suggest more active humoral responses in TRs (in line with their high responsiveness to vaccination), whereas NRs presented a consistently up-regulated inflammatory signature.

Assessing vaccination response and associated BTMs in TRs and NRs, we observed distinct postvaccination dynamics between TRs and NRs. TRs showed an early response at 3 days after vaccination, evident from a small proportion of DEGs, followed by the highest change in gene expression at day 7 before returning to prevaccination gene expression levels. However, NRs did not show statistically significant DEGs at any time point after vaccination compared to before vaccination (Fig. 3D). Functionally, the pathways enriched in comparison of day 3 versus day 0 pathways enriched were related to innate immune modules including antiviral, interferon, and complement activation (fig. S5C and table S8). While these pathways were only transiently up-regulated in TRs at 3 days after vaccination, NRs persistently up-regulated these pathways, along with other inflammatory/immune-related pathways (fig. S5D). Examining the genes and proteins enriched in the persistent inflammatory pathways, we identified PTX3, CST7, and HSPA1A up-regulated in NRs at prevaccination compared to TRs (fig. S5E). These three proteins have been well described for their role in inflammation and inflammatory diseases (*40*–*43*). These results highlight that while TRs mounted an immune response, evident from transcriptional changes at different time points (percentage of DEGs at day 3 and day 7), NRs maintained an inflammatory profile at all time points, which may impede mounting of the vaccine response.

### Nonresponders show higher activation of NK cells 7 days after vaccination

Examining the major changes in DEGs and BTMs at day 7 after vaccination in TRs and NRs separately (Fig. 3D), we found that DEGs identified in TRs were strongly positively enriched for multiple modules related to cell cycle, plasma cells, immunoglobulins (Igs), and T cell activation, among others, whereas in NRs no statistically significant DEGs were found at 7 days after vaccination. NRs showed comparably fewer enrichments related to cell cycle, T cell activation, and plasma cells 7 days after vaccination (fig. S6A). We next associated the enriched modules with cell proportions identified through transcriptome deconvolution. Cell cycle modules were strongly associated with plasma cells in TRs [false discovery rate (FDR) < 0.05] suggesting clonal expansion of plasma cells, whereas cell cycle modules in NRs did not show statistically significant associations with any cell subsets (Fig. 3E). Plasma cell proportions transiently increased at this time point for TRs, whereas this was not seen in NRs (fig. S4A and S4B). Previous studies have also shown that an increase in antibody-secreting cells at day 7 correlates positively with high antibody fold change (*44*). In NRs, activation modules were strongly positively associated with NK and CD8 T cells. We also observed similar associations in TRs; however, these associations were weaker than in NRs (Fig. 3F). The deconvolution results also show higher proportions of activated NK cells in NRs (fig. S4B). Flow cytometry on donors from the replication cohort (season 2014–2015) showed similar observations (fig. S6B). In addition, a statistically significant negative association of neutrophils with T cell activation BTMs in NRs suggests modulation of T cell activation by neutrophils. Last, in NRs, regulatory T cells (T_regs_) were negatively associated with immune activation BTM, which may suggest attempts at suppression of inflammation in response to vaccination. Together, the observed associations link cell subsets to the functional changes characterized through BTMs and highlight multiple differences in TRs and NRs after vaccination. Particularly, we observe higher NK cell proportions and higher activation of NK cells as a characteristic of the NRs within the aging population.

To validate the identified BTMs as correlates of vaccine response (distinguishing TRs from NRs), we examined the impact of genetic variation on antibody response across the discovery cohort (*N* = 176). Specifically, we used genome-wide quantitative trait loci (QTL) mapping, linking the serological response to vaccination (antibody log fold change per strain) to imputed genotypes using a linear regression model (see Materials and Methods and fig. S7A). This approach aligns with our previous cytokine QTL mapping studies (*45*). The BTMs that separated TRs and NRs (Fig. 3B) show suggestive enrichments in association with antibody QTLs (nominal *P* value <0.05; Fig. 3G), based on gene set analysis of MAGMA (*46*) (Materials and Methods; fig. S7B). Notably, these pathways were related to antibody production and inflammation, including plasma cell and myeloid modules. Upon examination of the ranked gene list associated with antibody QTLs through gene set analysis using MAGMA, it is noteworthy that the top-ranked genes align with those exhibiting statistically significant differential expression between TRs and NRs (Fig. 3H). Together, these results suggest transcriptome signatures for a differential vaccination response and their anchoring at the genetic level.

### Multiomics integration reveals a common axis of variation across layers separating HRs and LRs

To gain a holistic and nuanced perspective on the intricacies of the immune response upon vaccination, we performed an integrated analysis. First, we integrated plasma molecules and metabolites up-regulated at day 7 with transcriptome BTMs to derive their functional significance (fig. S8A). In TRs, protein concentrations of TNFRSF13B and IFNLR1 were strongly positively associated with B cells and plasma cell Ig modules from transcriptome (FDR < 0.05). The CCL3, GZMA, and CD48 protein concentrations were also statistically significantly positively associated with T cell transcriptional modules, suggesting their role in T cell activation. Among metabolites, uridine and tyrosine also showed positive associations with T cell activation and plasma cell numbers, which may imply their involvement in mounting the immune response. Uridine has previously been shown to be important for T cell proliferation (*47*). β-alanine, lactic acid, and serine all positively correlated with NK cell BTMs (fig. S8A). In contrast, no statistically significant associations between the proteome and transcriptional modules were found in NRs. Combining these results, we describe the potential significance of these proteins and metabolites in shaping the protective vaccination response in TRs.

**Fig. 4.**
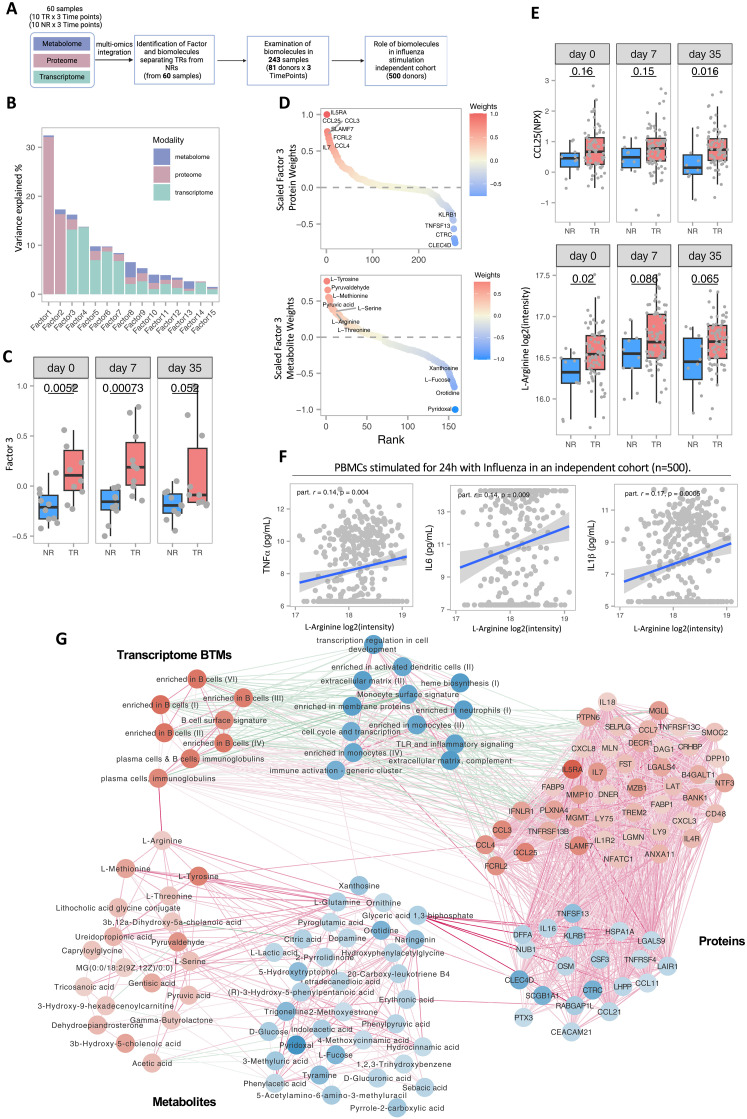
Multiomics integration of HRs and LRs. (**A**) Schematic overview of multimodal integration analysis. Schematic created using Biorender.com. (**B**) Barplot showing the proportion of explained variance per factor resulting from unsupervised factor analysis method MOFA. Colors within each bar indicate the contribution of each modality. (**C**) MOFA factor 3 separates TRs from NRs irrespective of time and across modalities. *Y* axis represents the factor 3 variance value attributed to each donor. *P* values are generated using a Wilcoxon rank sum test. (**D**) Top most positive and negative molecules within the metabolome and proteome modalities as calculated by MOFA. Molecules are ranked on MOFA scaled weights for factor 3. Positive weights are for TRs, whereas negative weights correspond to NRs. (**E**) Abundance of CCL25 and l-arginine in all 81 TRs and NRs. *P* values are generated using a Wilcoxon rank sum test. (**F**) Correlation of arginine with cytokine response after influenza stimulation in 500 FG cohorts. *P* values are generated using *t* test. Partial correlation estimate was corrected for age and gender. (**G**) Integrative network of transcriptome, proteome and metabolome data. Nodes in the network correspond to molecules (proteome and metabolome) or BTMs (transcriptome). Only molecules and pathways identified by MOFA weights (fig. S7E) are plotted. Edges in the network (all adjusted *P* < 0.05) are statistical associations from linear mixed models (see Materials and Methods), where red edges represent positive associations, and blue edges represent negative associations.

Next, we integrated the three omics layers to examine the interconnected processes underlying the heterogeneous influenza vaccine response in the aging population and examined their correlation to cytokine concentrations to stimulation, particularly for metabolites (Fig. 4A). Using multiomics factor analysis (MOFA) (*48*, *49*), an unsupervised method, we identified 15 latent factors that explained different levels of data variation along with varying contributions of omics layers within each latent factor (Fig. 4B). While the first two variables explained technical noise as estimated by MOFA, factor three explaining ~18% variance showed a common axis of variation across three omics layers. Factor 3 also separates TRs from NRs irrespective of time (Fig. 4C). Examining the significance of the other factors showed that factors 4 and 6 were consistent with the time dynamics differences observed in TRs and NRs (fig. S8B).

To assess the importance of this common axis (latent factor 3), we examined the top proteins and metabolites identified through this factor (Fig. 4D) and evaluated these in all TRs (*n* = 71) and NRs (*n* = 10). Among the proteins, CCL25 and CCL3 were consistently more abundant in TRs than NRs. Similarly, for metabolites, the amino acids arginine and methionine were more abundant in TRs (Fig. 4E and fig. S8C). To further assess the significance of these amino acids in immune function, we examined the association of amino acids with cytokine production capacity, particularly induced upon influenza stimulation in an independent cohort of 500 healthy individuals (*45*, *50*). Arginine and methionine showed statistically significant positive associations with interleukin 1β (IL-1β), IL-6, and tumor necrosis factor–α (TNFα) (Fig. 4F and fig. S8D). The increased abundance of these amino acids in TRs and their positive association with proinflammatory cytokine production may indicate their involvement in shaping the adaptive immune response to influenza vaccination via modulation of cytokine production.

Last, we created a network of statistically significant intra- and interomics connections by associating plasma molecules with transcriptome BTMs (Materials and Methods), overlaying the weights assigned for TRs (red) and NRs (blue) from MOFA latent factor 3 (Fig. 4G). Across omics layers, the transcriptome and proteome were highly connected, whereas the metabolome was less connected to the other two layers. The metabolite arginine was positively associated with TR-related BTMs involving plasma cells and Igs. Similarly, xanthosine was positively associated with immune activation BTMs, which was estimated as a predictor for NRs. Both xanthosine and orotidine are involved in nucleotide metabolism, which plays an important role in inflammation (*51*, *52*). We also observed strong positive associations of glyceric acid 1,3-bisphosphate/1,3-BPG with up to seven proteins that are predictors for NRs. In summary, we describe a common axis of variation in transcriptome, proteome, and metabolome that highlights the interconnected differences in influenza vaccine response in TRs and NRs within the aging population.

### Prevaccination high plasma IL-15 concentration negatively correlates with antibody response

**Fig. 5.**
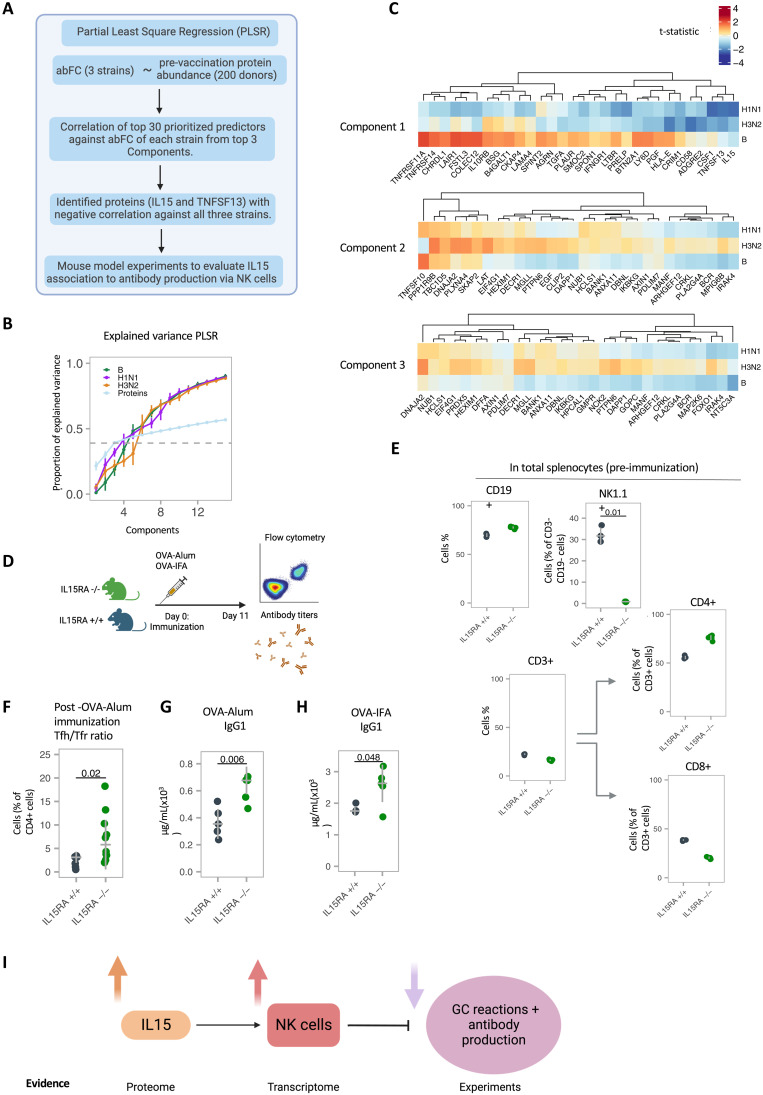
Prevaccination plasma proteome correlates to the antibody response to vaccination. (**A**) Schematic overview of analysis, created using BioRender.com. (**B**) Proportion of covariation in proteome and antibody fold change explained by each component for either each strain or the predictors examined through PLSR analysis with 10-fold cross validations (*N* = 200). The first three components using the predictors were able to explain ~40% covariation in the antibody response and prevaccination protein abundance. (**C**) Heatmap of *t* test statistics for top predictors with antibody fold change for top three components as calculated using rank product analysis for these components. IL-15 and TNFSF13 in component 1 both show negative association to all three antibody fold changes of all three strains. (**D**) IL15RA^+/+^ and IL15RA^−/−^ mice were immunized with OVA-Alum or OVA-IFA in the footpad, followed by analysis of antibody titers, and lymphocyte populations from inguinal lymph nodes (LNs) 11 days after immunization. (**E**) Proportion of B (CD19^+^), T (CD3^+^), NK (NK1.1^+^) cells, as well as CD4^+^ and CD8^+^ T cell subsets, among splenocytes of unimmunized IL15RA^+/+^ and IL15RA^−/−^ mice. (**F**) Ratio of T_FH_/Tfr cells, calculated from their frequency among total CD4^+^ cells, within LNs of immunized IL15RA^+/+^ and IL15RA^−/−^ mice. (**G**) Serum concentration of OVA-specific IgG1 in mice immunized with OVA-alum or (**H**) immunized with OVA-IFA. Pooled data from two independent experiments; *n* = 8 to 10. (**I**) Schematic summarizing the mechanism of IL-15–mediated activation of NK cells leading to suppression of GC responses and low production of antibody fold change. Schematic created using Biorender.com.

Seroconversion, here represented by fold change of HAI titers against each of the three vaccine strains, is widely used as the standardized immune correlate of protection against influenza infection (*53*, *54*). To prioritize the important prevaccination plasma proteins for antibody response to all three strains of influenza vaccine concurrently, we used partial least-squares regression (PLSR) in 200 donors (discovery cohort) (Fig. 5A). This allowed us to perform a systematic investigation of heterogeneity at the prevaccination time point (day 0). The resulting proteins from the top three PLSR components explain ~40% variance of the antibody fold change within the cohort (Fig. 5B). We evaluated the top 30 proteins from each of the three components to examine the directionality of the association with antibody response per strain (Fig. 5C). Overall, the majority of the prioritized proteins showed contrasting associations with the three strains. TNFSF13/APRIL and IL-15 were negatively associated with antibody fold changes for all three strains (Fig. 5C and fig. S9, A and B, left). Both these proteins have been described as proinflammatory cytokines and in the pathogenesis of influenza infection (*55*–*57*). In the replication cohort, we also found IL-15 negatively associated with high antibody changes for two strains, whereas this was not true for TNFSF13, perhaps because of the small effect size that is hard to replicate in a smaller cohort (fig. S9, A and B, left). We also observed increased *IL15* in NRs at prevaccination in transcriptome dataset (fig. S9B, right). Single-cell RNA sequencing on human peripheral blood mononuclear cells (PBMCs) showed that IL-15 is mainly expressed by monocytes (*58*) (fig. S9C). On the basis of these results, the contrasting relationship of the majority of prioritized proteins against the three strains may suggest a complex relationship between these proteins involved in complete protection against influenza infection. However, the consistent negative association of IL-15 with antibody response in both the discovery and replication cohort suggests a detrimental role in protection against influenza.

### High IL-15 concentration leads to reduced antibody titers through NK cell–mediated suppression of germinal center responses

IL-15 is important for NK cell proliferation and maturation (*59*–*61*). From bulk RNA sequencing of discovery cohort and flow cytometry results in the replication cohort, we observed higher frequencies of activated NK cells and total NK cell numbers in NRs (fig. S4A). NK cells have been described to suppress germinal center (GC) responses (*62*, *63*). We therefore examined the impact of perturbation of the IL-15–NK cell axis on antibody production with *IL15RA*-deficient mice. We immunized *IL15RA*^+/+^ and *IL15RA*^−/−^ mice with ovalbumin (OVA-IFA or OVA-Alum) to examine the resulting humoral response 11 days after immunization, at the time of maximum GC response in this animal model (*64*) (Fig. 5D). Before immunization, *IL15RA*^−/−^ mice showed a marked reduction of NK cells in the spleen (as anticipated, given the importance of IL-15 for NK cell numbers), without statistically significant differences on B cell populations but with slight differences on the frequency of CD4^+^ and CD8^+^ T cells (a population that can also be affected by IL-15 reduction) (Fig. 5E and fig. S9D). In the draining lymph node (LN), we observed a consistent increase of CD4 T cells, T follicular helper (T_FH_), and GC B cells in *IL15RA*^−/−^ mice. Furthermore, there was a decrease in T_regs_ and Tfr cells in *IL15RA*^−/−^ mice, although without reaching statistical significance (*P* values >0.05) (fig. S9, E and F). However, the T_FH_/T follicular regulatory (Tfr) ratio, critical in the regulation of GC responses (*64*, *65*), was statistically significantly higher in *IL15RA*^−/−^ mice (*P* value = 0.02, Wilcoxon rank sum test; Fig. 5F). In the same mice that had an increased T_FH_/Tfr ratio, we observed higher production of antibodies, across two different immunization protocols [*P* values = 0.006 (OVA-Alum) and 0.048 (OVA-IFA), Wilcoxon rank sum test; Fig. 5, G and H]. In conclusion, these results suggest that the reduction of IL-15 and NK cells led to more effective antibody production, in line with the data from aging population NRs who displayed higher IL-15 and NK cell activation together with poor antibody response (Fig. 5I).

### Prevaccination plasma malic and citric acid concentrations negatively correlate with antibody response

**Fig. 6.**
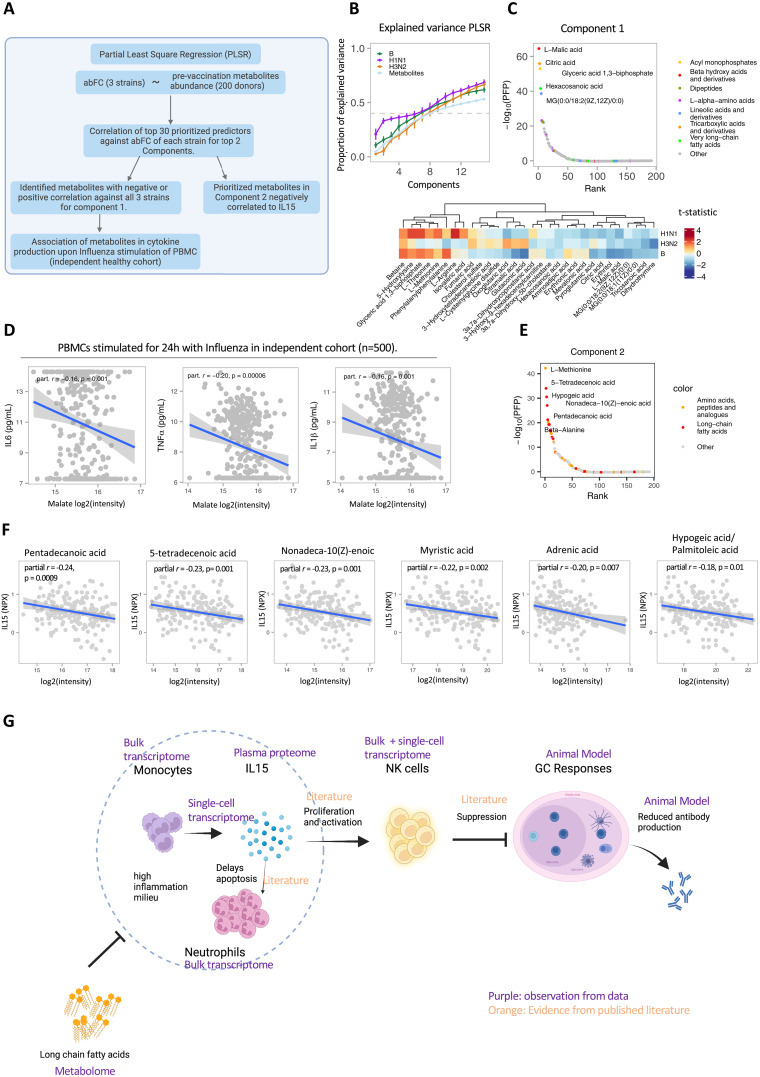
Prevaccination plasma metabolites as modulators for vaccination response. (**A**) Schematic overview of the analysis. (**B**) Proportion of covariation in endogenous metabolites and antibody fold change explained by each component for either each strain or the predictors examined through PLSR analysis with 10-fold cross validations (*N* = 200). The first eight components were able to explain ~40% covariation in the antibody response and prevaccination metabolite abundance. (**C**) Plot of metabolites from component 1 with their ranks and estimated percentage of false predictions (PFP) (top). Heatmap of *t* test statistic for the top predictors with antibody fold change for the top component as calculated using rank product analysis for these components (bottom). Malic acid and citric acid show negative correlation, while betaine shows positive correlation with antibody fold change against all three strains. (**D**) Association of malic acid to cytokine production upon influenza stimulation in an independent cohort of 500 younger healthy individuals, showing negative correlation. *P* values are generated using *t* test. Partial correlation estimate was corrected for age and gender. (**E**) Top metabolites for component 2 with their ranks and estimated percentage of false predictions show LCFAs as top candidates. (**F**) Negative correlation of these LCFAs identified in PLSR component 2 with IL-15. *P* values are generated using *t* test. Partial correlation estimate was calculated, corrected for age and gender. (**G**) Schematic describing the role of IL-15 in delayed neutrophil apoptosis, maturation and activation of NK cell populations, leading to suppression of GC responses and reduced antibody production and a chronic inflammatory status. Evidence for each observation is derived through different omics layers and experiments. Connections among different observations were also supported by published findings. Schematics created using Biorender.com.

To evaluate the prevaccination metabolite levels with antibody responses against three strains, we used PLSR (Fig. 6A). Herein, the metabolites in the top eight PLSR components explain ~40% variation of the antibody fold change among donors suggesting high interindividual variation in the donors reflecting on the metabolic differences (Fig. 6B). Metabolites prioritized in the first component showed malic acid and citric acid as top molecules, with negative associations to antibody response (Fig. 6C and fig. S10A). To derive the role of citric acid and malic acid in immune function, we associated prevaccination abundance of these metabolites with deeply phenotyped immune cell counts and cytokine production induced upon influenza stimulation in two independent cohorts of 300 and 500 healthy individuals, respectively (*12*, *45*, *50*, *66*). Citric acid showed negative associations with CD4^+^CCR6^+^CCR5^+^CCR7^+^ T cells along with three different subsets of immature neutrophils (FDR < 0.05) (fig. S10B). Malic acid showed strong negative associations with TNFα, IL-6, and IL-1β cytokine levels upon influenza stimulation (Fig. 6D). Furthermore, we also found betaine as one of the top predictors in component one, positively associated with antibody response across all strains (Fig. 6C and fig. S10C). Betaine has been described for its inhibition of IL-1β production and release (*67*, *68*) and also for its suppression of proinflammatory signaling during aging (*69*). These results reflect the impact of prevaccination metabolite abundance linked to different physiological immune states across responders culminating in differential vaccination response in the aging population.

### Unsaturated long-chain fatty acids are negatively associated with prevaccination IL-15 abundance

Given the putative role of high IL-15 in suppressing antibody production in the aging population, we evaluated the relationship of prevaccination IL-15 abundance and metabolites abundance. Prevaccination IL-15 showed strong negative associations with odd-chain (pentadecanoic acid) and certain unsaturated long-chain fatty acids (LCFAs; e.g., palmitoleic acid) (*P* value <0.02) (Fig. 6F and fig. S10D). These LCFAs were also among the top 30 predictors for PLSR component 2, explaining variation in antibody fold change among the donors (Fig. 6, E and F). LCFAs and especially polyunsaturated fatty acids have been described as important immunomodulatory metabolites to protect against infections (*70*). To test the significance, particularly of LCFAs in suppressing inflammatory proteins, we examined the relationship of all prevaccination lipid and lipid-like molecules (taxonomy class) with these proteins. LCFAs were negatively associated with inflammatory proteins, whereas other lipid molecules showed both positive and negative associations (fig. S10E). We also examined the role of LCFA in a younger cohort [500FG (*45*), with 98% donors less than 65 years of age]; however, the LCFA-specific negative associations with inflammatory proteins were absent (fig. S10E). Among the LCFAs, the most statistically significant negatively associated metabolite was pentadecanoic acid, an odd-chain fatty acid with anti-inflammatory properties and resistance to oxidation (*71*, *72*). Together, the data suggest an age-dependent role of LCFAs in the control of inflammation. Together, we describe certain fatty acyls negatively associated with IL-15 and also their association with the antibody fold change. This indicates toward the role of metabolites in modulating the vaccine response in the aging population, particularly LCFAs as potential prevaccination suppressors of high inflammation.

## DISCUSSION

In this work, we evaluated a large cohort of aging individuals to understand the heterogeneity of influenza vaccination response, specifically to address two main questions: (i) How does the vaccination response differ between responders and nonresponders? and (ii) Which prevaccination biomarkers correlate with the vaccination response (fig. S1A)? We examined the transcriptome, proteome, and untargeted metabolome of these donors separately, as well as across omics layers and found postvaccination dynamics that were indicative of mounting of sufficient immune response to vaccination in responders (HRs and TRs), whereas these dynamics were absent or altered in NRs and LRs. Plasma proteome changes upon vaccination show consistently up-regulated proteins in both HRs and TRs, suggesting crucial role of these proteins in mounting the immune response. Additional proteins were up-regulated at day 7 in TRs including HLA-E and SIGLEC10 known for their suppressive role in inflammation (*26*–*28*), while CD48 (*25*) involved in lymphocyte activate suggests an optimum shaping of the immune response to vaccination. We also examined whether the prevaccination protein abundance, particularly those proteins implicated in vaccine response, were high enough in NRs and therefore cannot be further increased upon vaccination. However, this was not the case, as both TRs and NRs had comparable levels of these proteins at prevaccination. Plasma metabolome changes upon vaccination show up-regulation of amino acids (*35*) and down-regulation of fatty acids in HRs and TRs. In particular, significant up-regulation of vitamin B6 (*23*) metabolite in HRs, missed in LRs, and missed up-regulation of amino acids in NRs suggest differential metabolic regulation in these donors and may suggest a lack of mounting of the immune response in LRs.

At transcriptome level, NRs were characterized by increased and activated NK cell populations and persistent inflammation from the discovery cohort, while flow cytometry results from replication cohort also agreed with this finding. In agreement, the NK cell receptor *KLRB1* was shown as a negative predictor for vaccine response in the elderly (*17*), whereas *NKG2C* expression was reported as positively correlated with influenza vaccination (*73*) in healthy individuals. This opposing observation of NK cells may be related to the aging immune system. Signaling through inflammatory molecules is important for mounting an immune response, while persistent low-grade inflammation, termed “inflammaging,” can result in exhaustion and is detrimental to the vaccine response (*74*). In NRs, evidence from the transcriptome suggests persistent inflammation driven through AP1 transcriptional networks, among others. Using multiomics integration for day 7 and across time points, we show key molecules that are important in supporting and leading to a productive vaccine response. Some of these identified molecules e.g., CCL25, CCL3, and arginine, have already been studied as potential immune adjuvants in vaccination and/or as supplementation for a better humoral response to vaccination (*75*–*78*). CCL25, a chemokine ligand for CCR9, has been shown to play both pro- and anti-inflammatory roles in various diseases (*79*), while CCL3 and its receptor CCR5 involvement in leukocyte recruitment into the airway to up-regulate antiviral responses in influenza infection (*80*). Amino acids in general are crucial for immune function (*35*, *81*), and we show functional associations of plasma arginine and methionine to cytokine production upon influenza stimulation using an independent cohort. In summary, we indicate biomarkers that support both a robust immune response to the vaccine response and influenza stimulation.

We propose a model where high abundance of prevaccination IL-15 leads to increased activated NK cells, which in turn inhibit the GCs to suppress antibody production (Fig. 6F). Although the murine experiments used a model protein and adjuvants, instead of an influenza vaccine, the observations remain consistent with this model. Modulation of adaptive immune responses through NK cells has been proposed for better vaccine design, where NK cells control the magnitude and quality of immune response (*82*–*84*). Previous work and our current results have shown lower levels of T and B cells in NRs compared to TRs (*7*). The simultaneous decrease of T and B cells, with increased NK cell proportions, may further lead to suppression of antibody responses in NRs. Monocytes are the main source of IL-15 in immune cell populations in human blood and are reported to express more IL-15 with increasing age (*85*). We did not observe higher monocyte proportions but higher monocyte (inflammatory) transcriptional activity in NRs. IL-15 has also been described to inhibit apoptosis of neutrophils (*86*), which were more abundant in our NRs. Together, these findings suggest contributions from inflammatory monocytes and neutrophils (*86*) in maintaining a persistent inflammatory milieu in NRs. IL-15 is also up-regulated in TRs and HRs 35 days after vaccination. Therefore, particularly the high prevaccination IL-15 has a negative effect to vaccine response, while vaccination-induced IL-15 may indicate toward vaccine response through other cell subsets. For example, the receptor for IL-15, IL-15RA, is also associated with TRs in the multiomics integration. IL-15RA is expressed by dendritic cells, and the importance of the cross-talk between NK cells and dendritic cells in immune cell activation and maturation has been established (*87*). In addition, elevated IL-15RA in TRs may suggest dendritic cells–driven activation of T and B cells in response to vaccination. We did not observe differences in deconvoluted dendritic cell proportions between TRs and NRs. However, cross-talk between these cell subsets within the secondary lymphoid tissues could play a role during influenza infection, and its role in vaccination in the aging population is of interest for future research. Recent work has also highlighted likely role of IL-15 in monocytes, along with CD8 T cells, in COVID-19–recovered males leading to higher IFNG and increased in antibody responses after influenza vaccination (*88*). In our study, we also observed increased CD8 T cells activation in both TRs and NRs 7 days after vaccination; however, no differences were observed in the proportion of CD8 T cells between TRs and NRs. A recent study examining the transcriptome profiles in healthy adults under 50 years old), found that a prevaccination high inflammatory status is favorable for a strong vaccine response. This finding however, was not replicated for older populations. Our research indicates that a transient up-regulation of inflammatory molecules that promotes antiviral and interferon responses leads to a productive vaccination response, whereas a persistent low-grade up-regulation of inflammatory molecules does not. The top predictors from the metabolome PLSR component 2 consisted of methionine and fatty acids, particularly unsaturated LCFAs. Methionine was one of the top predictors for TRs picked by MOFA, as well as one of the top metabolites up-regulated at day 7 after vaccination in TRs. Unsaturated LCFAs were all negatively associated with IL-15. Recent work has highlighted the role of fatty acid metabolism in the IgG1 response to influenza (*11*). In line with this, we observed consistent down-regulation of fatty acyls in TRs after vaccination, whereas this only occurred in NRs at the later time point (day 35). These findings suggest a multifaceted role of fatty acyls by serving as energy molecules and/or signaling molecules, thereby modulating the immune response. Combining the insights from proteome and transcriptome, we provide a potential link of how chronic low-grade inflammation may lead to poor vaccine response and propose polyunsaturated LCFAs and odd-chain fatty acids to alleviate the chronic inflammation, leading to a more productive vaccination response in the aging population.

We also acknowledge the limitations of this study. Larger cohorts are required in future studies to fully explore the molecular mechanisms leading to a protective vaccine response. The role of comorbidities frequently observed in the aging population (type 2 diabetes, cardiovascular diseases, etc.) also affects the response to vaccination (*15*, *89*, *90*). Although inflammaging associated with proinflammatory cytokines is also emerging as a common feature of poor vaccine responsiveness in people with chronic diseases and old age, it will be critical to understand the potential universal character of the identified mechanisms and biomarkers by performing studies using other vaccines and populations. Therefore, future studies incorporating these factors will provide an improved understanding of the vaccine response in the aging population. Furthermore, in vivo experiments and clinical trials are required to assess whether modulation of metabolites can improve the vaccine response in (aging population) nonresponders.

In conclusion, we provide a comprehensive view of vaccine response differences within the aging population, implications of high prevaccination plasma IL-15, and its role in suppressing antibody production. We show the key molecular differences between HRs and LRs within the aging population across multiple layers and highlight several molecules that open avenues of research toward better vaccine design as well as boosting vaccine response.

## MATERIALS AND METHODS

The performed study complies with all relevant ethical regulations. The execution of the study as well as the use of the sampled human material was approved by the ethics committee (ref no. 6775, dated 15 September 2015) of Hannover Medical School. The documentation of all findings adheres to the Strengthening Reporting of Observational Studies in Epidemiology reporting guideline for cohort studies.

### Study population

The study population has been described before in detail by Riese, Akmatov, and colleagues (*7*, *13*). Briefly, a prospective population-based study across two influenza seasons (2014/2015 and 2015/2016) was performed among aging population individuals (>65 years of age) from Hannover, Germany. Donors from both seasons did not overlap. Donors from 2014/2015 influenza season were recruited as a part of pilot study cohort, which therefore consists of smaller sample size. Participants were recruited from a random sample from the local population registry and represented the general population as verified by a survey (*14*). Intravenous blood samples were drawn before vaccination (day 0) and on day 1, day 3, day 6/7, day 21, and day 70 after vaccination. Hemagglutination inhibition titers and MN titers were measured as described before (*7*). Briefly, we followed the formulation of the Fluad vaccine and used the following antigens for titer measurements in serum: H1N1 A/California/7/09 NYMC-X181 (both seasons), H3N2 A/Texas/50/2012 NYMC-223 (season 2014/2015), H3N2 A/Switzerland/9715293/2013 NIB88 (season 2015/2016), B/Massachusetts/02/2012 NYMC BX-51B (season 2014/2015), and B\Brisbane/9/2014 (season 2015/2016). Seroconversion was defined as either a postvaccination titer of ≥40 for individuals with prevaccination titers <10, or a fourfold increase in titer upon vaccination for individuals with prevaccination titer ≥10. Individuals were classified as TRs (seroconversion against all three strains), NRs (no seroconversion against any strain), or Other (seroconversion against at least one strain but not all three).

### Bulk transcriptome sequencing

Bulk transcriptome sequencing has been described before (*7*). In short, 10 TRs and 10 NRs were selected on the basis of their serological response to vaccination. We included five time points per individual: baseline, days 1/3, day 6/7, day 21/35, and day 60/70. MicroRNA (miRNA) and total RNA were purified from whole blood samples frozen in PAXgene tubes (BD) using the “PAXgene Blood miRNA Kit” (Qiagen). The detailed procedure was previously described (*7*).

### Untargeted metabolomics

We assessed the metabolic profiles of 702 samples (repeat measurements at three time points for 234 individuals) from two influenza seasons. Plasma samples were randomized across plates with respect to vaccine response, sampling time point, and sex. Polar metabolites were extracted from each sample with 180 μl of 80% methanol in a deep well extraction plate using 20 μl of plasma by General Metabolics (Boston, USA). Samples were vortexed for 15 s, incubated at 4°C for 1 hour, and centrifuged at 4°C, 3750 rpm for 30 min. Metabolome profiles of the sample extracts were acquired using flow-injection mass spectrometry. The method described here is adapted from previously described methods (*91*). The instrumentation consisted of an Agilent 6550 iFunnel LC-MS quadrupole orthogonal acceleration–time-of-flight mass spectrometer in tandem with an MPS3 autosampler (Gerstel) and an Agilent 1260 Infinity II quaternary pump. The running buffer was 60% isopropanol in water (v/v) buffered with 1 mM ammonium fluoride. Hexakis (1H, 1H, 3H-tetrafluoropropoxy)-phosphazene) (Agilent) and 3-amino-1-propanesulfonic acid (HOT) (Sigma-Aldrich). The isocratic flow rate was set to 0.150 ml/min. The instrument was run in 4-GHz high resolution, negative ionization mode. Mass spectra between 50 and 1000 mass/charge ratios were collected in profile mode. Each sample (5 μl) was injected twice, consecutively, within 0.96 min to serve as technical replicates. The pooled study sample was injected periodically throughout the batch. Samples were acquired randomly within plates.

### Targeted metabolomics

Serum concentrations of amino acids were determined by mass spectrometry, using a high-performance liquid chromatography–coupled triple quadrupole mass spectrometer (AP4000, Siex) and the AbsoluteIDQ p180 kit (Biocrates Life Science AG, Innsbruck, Austria) following the manufacturer’s protocols.

### Targeted proteomics

We measured 384 circulating proteins in plasma using Olink’s proximity extension assay (PEA) Explore Inflammation panel (*92*) in 702 samples (repeat measurements at three time points for 234 individuals). In the PEA, oligonucleotide-labeled antibodies (“probes”) bind the protein of interest. Linking of the probes is triggered by close proximity of two antibodies that limits cross-reactivity. Upon linking, the probe sequence is hybridized and subject to extension by DNA polymerases. The resulting sequence is quantified by real-time polymerase chain reaction. Protein values are expressed as normalized protein expression values, a relative value on a log_2_ scale. Quality control of the raw data was performed by Olink (incubation controls, extension controls, and detection controls).

### Immunization, cell isolation, and blood serum collection of *IL15RA*^−/−^ and *IL15RA*^+/+^ mice

Sex-matched *IL15RA*^−/−^ and *IL15RA*^+/+^ C57BL/6 mice, aged from 10 to 12 weeks, were immunized in the back paw footpad with OVA (Ovalbumin EndoFit, Invivogen, no. vac-pova) emulsified 1:1 (v:v) with either Incomplete Freund’s adjuvant (IFA, Sigma-Aldrich, no. F5506) or Alum (Alu-Gel-S, Serva, no. 12261–01). Each animal was inoculated with a volume of 50 μl per paw containing 80 μg of OVA (OVA-IFA was injected in two paws, while OVA-Alum was injected in one paw per mouse). Mice were euthanized 11 days after immunization and the cells from the spleen and inguinal LNs isolated. Blood serum from each mouse was also collected at the same time point by cardiac puncture. The mice were bred and maintained under specific pathogen–free conditions at the Instituto de Medicina Molecular (iMM), where the experiments were performed under an animal experimentation authorization granted by Direção-Geral de Alimentação e Veterinária, the Portuguese national authority for animal health, and iMM-ORBEA, the Ethic Committee for laboratory animal care at the iMM Rodent Facility in Lisbon.

### Flow cytometry of *IL15RA*^−/−^ and *IL15RA*^+/+^ mice

Characterization of the lymphoid cell populations in the spleen of *IL15RA*^−/−^ and *IL15RA*^+/+^ C57BL/6 strains was done by flow cytometry with the following antibodies: anti–CD19–fluorescein isothiocyanate (FITC) (MB19-1, eBioscience; 1:100), anti–CD3–allophycocyanin (APC) (145-2C11, eBioscience; 1:100), anti–CD4-BV510 (RM4-5, BioLegend; 1:400), anti–CD8-eF780 (53-6.7, eBioscience; 1:200), and anti–NK1.1-PECy7 (PK136, eBioscience; 1:100). B and T cells subpopulations present in the inguinal LNs were first preincubated with anti–CXCR5-Biotin (2G8, BD Biosciences; 1:50) and subsequently stained with anti–CD19-FITC (MB19-1, eBioscience; 1:100), anti–CD95-phycoerythrin (Jo2, BD Biosciences; 1:100), anti–Inducible T cell costimulator (ICOS)-PerCPCy5.5 (7E.17G9, BioLegend; 1:200), anti–PD-1-PECy7 (J43, eBioscience; 1:100), anti–GL7-eF660 (GL-7, eBioscience; 1:100), anti–CD25-APCeF780 (PC61.5, eBioscience; 1:200), anti–CD4-BV510 (RM4-5, BioLegend; 1:400), and anti–CD44-BV605 (IM7, BioLegend; 1:400) together with Streptavidin-BV711 (BioLegend; 1:100). Intracellular Foxp3 (FJK-16 s, eBioscience; 1:100) staining was performed using the Foxp3 Fix/Perm Kit (eBioscience, no. 00-5521-00) according to the manufacturer’s instructions. The samples were acquired on a BD LSRFortessa cytometer from the iMM Flow Cytometry Facility and further analyzed on FlowJo software (TreeStar).

### Anti-OVA IgG quantification of *IL15RA*^−/−^ and *IL15RA*^+/+^ *mice*

Serum anti-OVA mouse IgG1 was quantified by enzyme-linked immunosorbent assay (ELISA). Ninety-six–well plate wells were coated with 50 μl of OVA (Ovalbumin EndoFit, Invivogen, no. vac-pova) at 20 μg/ml in phosphate-buffered saline (PBS) overnight (4°C), washed three times with ELISA Buffer (Invitrogen, no. 88-50620-88), and subsequently blocked with 200 μl of ELISA Buffer for at least 30 min. After washing, standards and different dilutions of each serum sample were plated in duplicate in the coated wells for at least 90 min. The standard curve was obtained with measurements from known concentrations of anti-OVA [6C8] IgG1 antibody (AbCam, no. ab17293). After washing, 50 μl of anti–mouse-IgG1–horseradish peroxidase (SouthernBiotech, no. 1070-05) diluted 1:2000 was added to the wells and incubated for at least 45 min. Wells were washed, and 50 μl of the substrate solution 3,3′,5,5′-tetramethylbenzidine (TMB Single Solution, Life Technologies) was added per well. The reaction was stopped with 25 μl of sulfuric acid (H_2_SO_4_) according to the color development of the standards. Last, optical density (OD) was read at 450 nm within 30 s after stopping the reaction. Quantification of anti-OVA mouse IgG1 in each sample of blood serum was attained by calculating the concentration of antibody considering the OD values obtained for a dilution that fitted within the OD values of the standard curve (0.2 > OD < 1).

### Flow cytometry of human PBMCs

Cryopreserved PBMCs were thawed, rested for 2 hours and incubated overnight with purified anti-human CD28 and CD49d at a concentration of 10 μg/ml. For surface staining, cells were incubated with the antibody mixture prepared in PBS for 30 min at 4°C in the dark. Cells were stained for flow cytometric analysis using the following antibodies: CD3 (BUV395, SK7, catalog no. 564001, dilution 1:200; BD, Franklin Lakes, New Jersey, USA) and CD56 (BV785, 5.1H11, catalog no. 362550, dilution 1:100; BioLegend, San Diego, USA). The samples were acquired at a BD Fortessa flow cytometer using the Diva software and analyzed using FlowJo.

### Statistical analyses

#### Bulk transcriptome analysis

We quantified bulk RNA sequencing data using Salmon (*93*) using default parameters and the human GRCh38 genome. To check for potential outliers, we performed principal components analysis (PCA). No samples were removed on the basis of this analysis. We then imported the transcript-level quantifications and transcript lengths using tximport (*94*) and quantified the differential expression between responder groups and over time points using a limma-voom approach (*95*, *96*): *gene* ~ *responder + age + Time + sex + 1|donor*. For time dynamics, following linear model was used: *gene ~ responderTime + age + sex + 1|donor*. Functional enrichment was performed using GSEA using BTMs. Genes were ranked by *t* test statistic from this mixed linear model for the comparison of interest. *P* values were adjusted using Benjamini-Hochberg, where we considered adj. *P* value <0.01 significant or adj. *P* value <0.05.

The relative contribution of individual cell types was estimated using CIBERSORT using the LMM22 background. We defined the pathway activity as the mean expression corrected for variance across donors of all genes assigned to a specific BTM per sample. A linear model was used to associate the estimated proportions of individual cell types to functional pathways: *cell proportion ~ BTM_score + age + sex + 1|donor*, done separately for TRs and NRs. where *BTM_score* is the BTM score as described above and *cell proportion* is the estimated cellular proportions from CIBERSORT per individual. *P* values were adjusted on all associations using Benhamini-Hochberg. We considered adj. *P* values <0.05 to be significant.

#### Genotype imputation and quality control

Genotyping was carried out using an Illumina Infinium Global Screening Array, yielding genotypes for 176 individuals. We verified that the genotyped sex matched the reported sex for each donor. We discarded no samples based on heterozygosity (maximum 3 SDs from the mean) or missingness (maximum 3% missingness). After these quality control steps, genotypes were imputed using the Michigan imputation server against the HRC1.1 2016 as the reference panel. Eagle v2.454 was used for phasing. After imputation, we removed variants with *R*^2^ < 0.5, minor allele frequency < 0.01, Hardy-Weinberg equilibrium *P* value < 1 × 10^−12^. After filtering, we retained 54,147,97 single-nucleotide polymorphisms (SNPs) from 176 individuals for further QTL mapping.

#### Antibody QTL mapping and MAGMA analyses

We mapped the serological response to vaccination (that is, the log fold change in HAI titers upon vaccination). Values were rank-normalized before mapping. Age and sex were included in the linear models as covariates. Using these antibody QTL results, we used MAGMA v1.10 (*46*) to collapse variant-level *P* values into gene-level *P* values using the standard snp-wise model. We used windows of 0.5 kb on either side of a gene to assign variants to genes. The 1000G data was used as reference for linkage disequilibrium by MAGMA. Next, we used the BTMs to collapse gene-level *P* values into *P* values for transcriptional modules.

#### Targeted proteomics

We removed measurements that were flagged as unreliable by Olink as well as protein assays where the target protein was detected in less than 70% of the samples. Last, we considered 311 high-quality proteome assays for further downstream analyses. We performed dimensionality reduction (PCA) to check for potential outliers or batch effects and did not identify either.

We assessed the proteomic differences between HRs and LRs per strain and time point, as well as the differences over time for HRs and LRs separately using a linear mixed model: *protein ~ strainresponse_time + age + sex + 1|donor*, where strainresponse_time is a combined factor indicating the serological response to a strain (either high or low) and the time.

To quantify the differences in protein abundances between time points within each responder group separately, we fit a linear mixed model using limma: *protein* ~ *time_responder* + age + *sex* + *1|donor*, where *time_responder* indicates the sampling time point and responder group, and *donor* is the random effect.

For all models, Benjamini-Hochberg post hoc correction was used to control the FDR across all proteins. We considered adjusted *P* values <0.05 significant.

### Untargeted metabolomics

Raw metabolite profile data were centroided, merged, and recalibrated using MATLAB software as previously described. Putative annotations were generated on the basis of compounds annotated in the Human Metabolome Database (HMDB) database using both accurate mass per charge and isotopic correlation patterns. We retained only ions that were confidently annotated, allowing 0.001-Da tolerance between the ion and its corresponding annotation. Because exogenous and/or drug-related metabolites do not reflect an individual’s current immune status and could lead to possibly confounding effects, we aimed to consider a set of endogenous metabolites. To do so, considered endogenous metabolites from HMDB and matched this to our annotated metabolite data based on the ion’s chemical formula. Then, we manually rechecked whether metabolites were drug related using the DrugBank database. Metabolites associated with medications or other xenobiotics were removed. Last, we retained 192 endogenous metabolites for further analyses.

We validated the quality of our untargeted metabolome data by using a targeted metabolomic approach on a subset of the individuals. We manually correlated 18 primary amino acids that were reliably detected and annotated between the two platforms and observed good replicability (median Pearson’s *r* = 0.77). This indicates good sample quality and reliable, consistent measurements (fig. S11).

We assessed the endogenous metabolic differences between HRs and LRs per strain and time point, as well as the differences over time for HRs and LRs separately using a linear mixed model: *metabolite ~ strainresponse_time + age + sex + 1|donor*, where *strainresponse_time* is a combined factor indicating the serological response to a strain (either high or low) and the time.

To quantify differences in endogenous metabolites within responder groups (that is, TRs and NRs) over time, we used the model: *metabolite* ~ *time_responder* + age + *sex* + *1|donor*, where *time_responder* indicates the sampling time point and responder group, and *donor* is the random effect.

For all models, we applied Benjamini-Hochberg post hoc correction on all endogenous metabolites and considered metabolites with adjusted *P* values <0.05 significant. Metabolite classifications to annotate metabolites were retrieved from HMDB. We performed metabolite enrichment by performing over-representation analysis using IMPALA. Adjusted *P* values (Benjamini-Hochberg) < 0.05 were considered significant.

### Integration of transcriptome, proteome and, metabolome layers

We aimed to provide a comprehensive integration of the different data layers that were generated. To do so, we used two approaches. First, we associated circulating protein and metabolite levels to transcriptome pathway activity. We defined pathway activity as described above. Proteins and metabolites were selected when differentially abundant at day 7 compared to day 0. Associations were estimated using a linear mixed model: *protein ~ age + gender + BTM + 1|donor. P* values were adjusted using Benjamini-Hochberg over all obtained associations, and adjusted *P* values <0.05 were considered significant. We performed this analysis for day 7 as we observe the peak of the adaptive immune response to vaccination on this day.

Next, we used MOFA (*48*, *49*) to perform unsupervised integration of all layers. We chose day 0, day 7, and day 35 as we had datasets for these time points across all layers for the highest (*N* = 10) and lowest (*N* = 10) responders. For transcriptome, the top highly variable genes were selected. For proteome and metabolome, we removed molecules that were significantly (nominal *P* value <0.05) associated with sex. Last, we considered 1560 genes, 279 proteins, and 158 metabolites across three time points for further analysis using MOFA. We applied MOFA standard practices and trained the model on a single group. The default data and training model options were used except for scale_views set to TRUE. Following model training, MOFA reported factor 1 correlated with technical variation, and we thus did not consider this factor. The rest of the factors were evaluated for significance with either Responder Category, Age, Gender, and Time by testing the factor values using a Wilcoxon rank sum test. For the factors that are shown, weights are scaled for each layer independently and used to identify the top predictors for proteome and metabolome. For network building, we performed GSEA using BTMs on the transcripts ranked by MOFA’s scaled weights and considered adjusted *P* values <0.05 significant. For proteome and metabolome, molecules with absolute scaled weights >0.25 for factor 3 were considered and their associations done, similar to BTMs. Associations across layers of protein and metabolite molecules to different BTMs controlling for repeated measurements from the same donors were done and associations with adjusted *P* values <0.05 were selected. Across-layers associations were also calculated and significant associations were kept. Cytoscape (*97*) was used to build the multilayer network with color of the nodes representing factor 3 scaled weights for each protein or metabolite, or enrichment score for transcriptome BTMs.

### Partial least-squares regression

We used PLSR to identify the prevaccination proteins and metabolites that are most consistently associated with the serological response against the three influenza strains. PLSR was performed using the *pls* library in R for proteome and metabolome separately using all 200 donors of the discovery cohort. The model was run for 100 independent iterations with 10-fold cross-validations (e.g., 90% training, 10% testing), and we calculated the proportion of variation explained by each PLSR component. For the first three components, we performed rank-product analysis over the 100 iterations to identify the top predictors. The top 30 ranked molecules (either proteins or metabolites) were then evaluated for their positive or negative association with antibody fold change.
