# Supplementary material for: Systemic dysregulation and molecular insights into poor influenza vaccine response in the aging population
Source: Sci Adv. 2024 Sep 27;10(39):eadq7006. doi: 10.1126/sciadv.adq7006 (PMC11430404; doi:10.1126/sciadv.adq7006)
Supplement: Supplementary file 1 — Figs. S1 to S11 Legends for tables S1 to S8 [file sciadv.adq7006_sm.pdf]

Science Advances
AAAS

Supplementary Materials for

Systemic dysregulation and molecular insights into poor influenza vaccine response in the aging population

Saumya Kumar *et al.*

Corresponding author: Yang Li, yang.li@helmholtz-hzi.de

Sci. Adv. **10**, eadq7006 (2024)
DOI: 10.1126/sciadv.adq7006

The PDF file includes:

Figs. S1 to S11
Legends for tables S1 to S8

Other Supplementary Material for this manuscript includes the following:

Tables S1 to S8

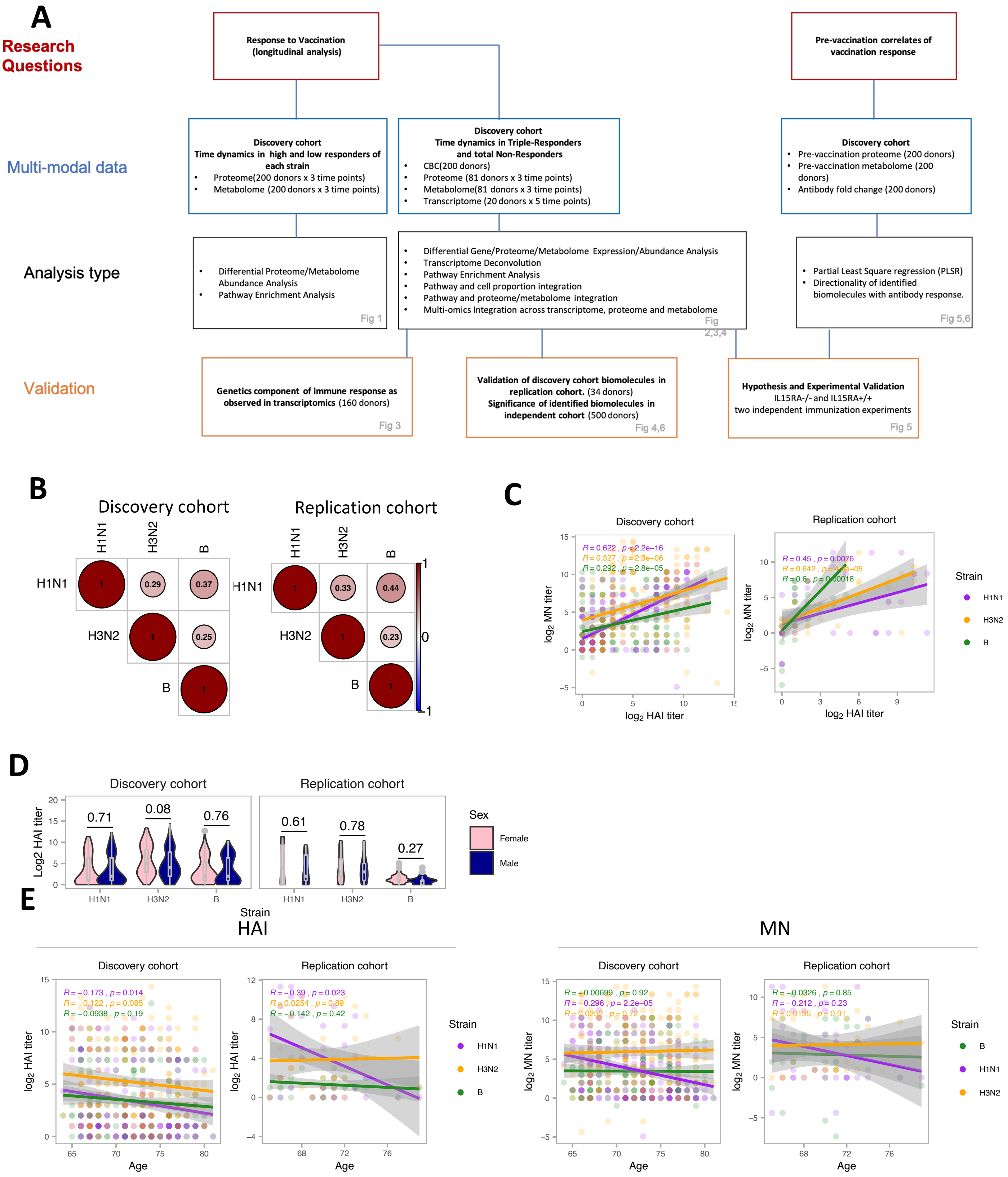

Figure S1. Cohort, datasets, analysis outline and general demographic factors associated with the serological response to trivalent inactivated influenza vaccination. (A) Structured outline of datasets used, different comparisons examined and validations **(B)** Correlation among hemagglutination (HAI) titres fold-change upon vaccination. In both seasons, HAI titres against each of the three strains are moderately positively correlated to each other. **(C)** Correlation between MN titres and HAI titres across two influenza seasons. Each dot is a sample, colours represent influenza strains. **(D)** Sex dependant differences in serological response to TIV across two seasons. P-values were generated using the Wilcoxon ranked-sum test. **(E)** Influence of age on the HAI and microneutralization (MN) titres. Each dot is an individual, colours represent the different influenza strains.

Figure S1

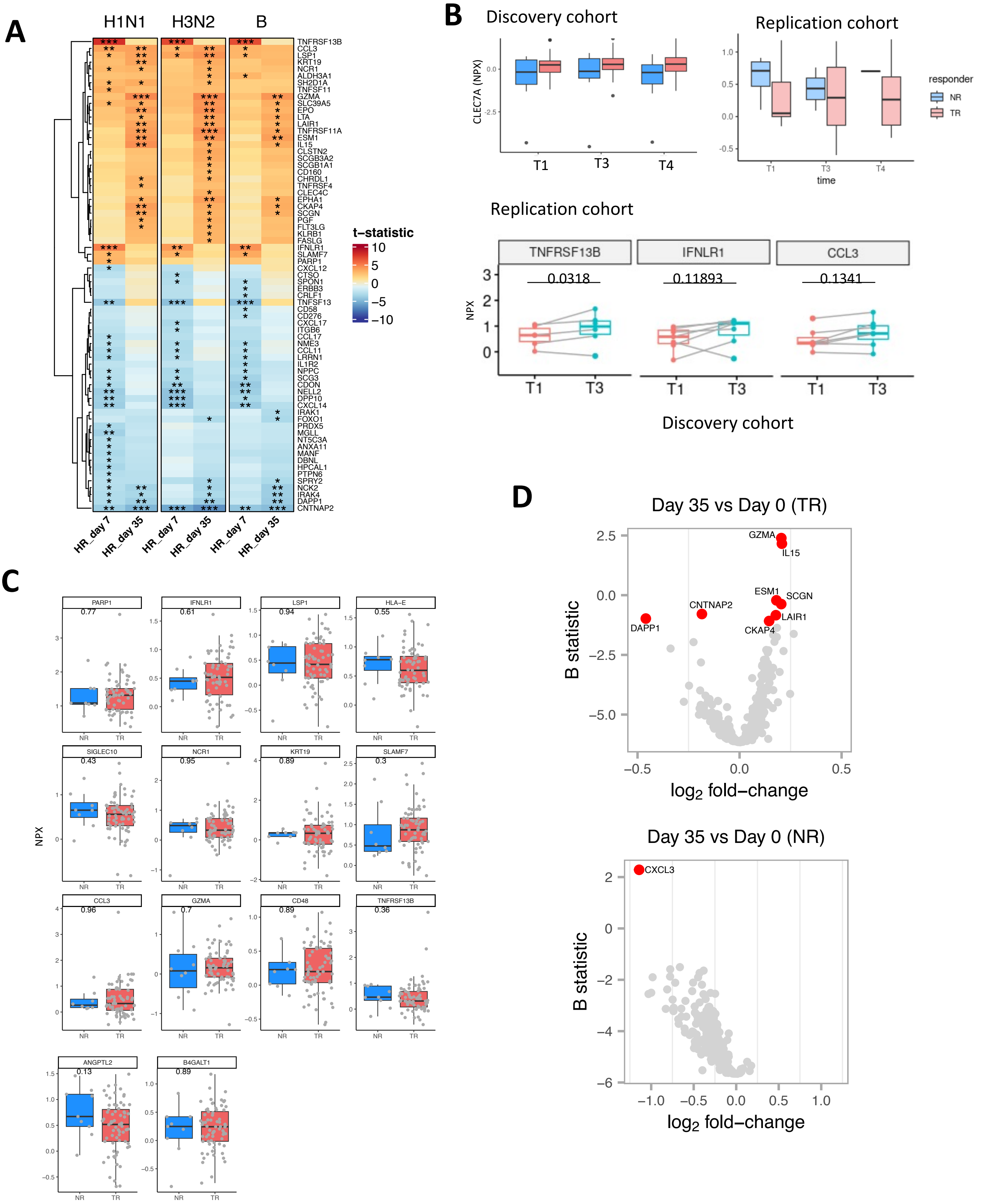

Figure S2. Proteomic response to TIV (A) Heatmap showing persistent changes in HRs across all 3 strains in response to vaccination **(B)** CLECL7A proteome abundance in all TRs (red) and NRs (blue) over time in the Discovery cohort and the Replication cohort (top). Proteome differential abundance results in the replication cohort (bottom). (**C)** Boxplot of pre-vaccination protein levels in TRs and NRs. These proteins were upregulated in TRs at day 7 post-vaccination. **(D)** Volcano plots showing differential abundance results comparing Day 35 vs Day 0 for TRs (top) and NRs (bottom) in discovery cohort. Red dots represent significantly different proteins at adjusted p-value < 0.05.

Figure S2

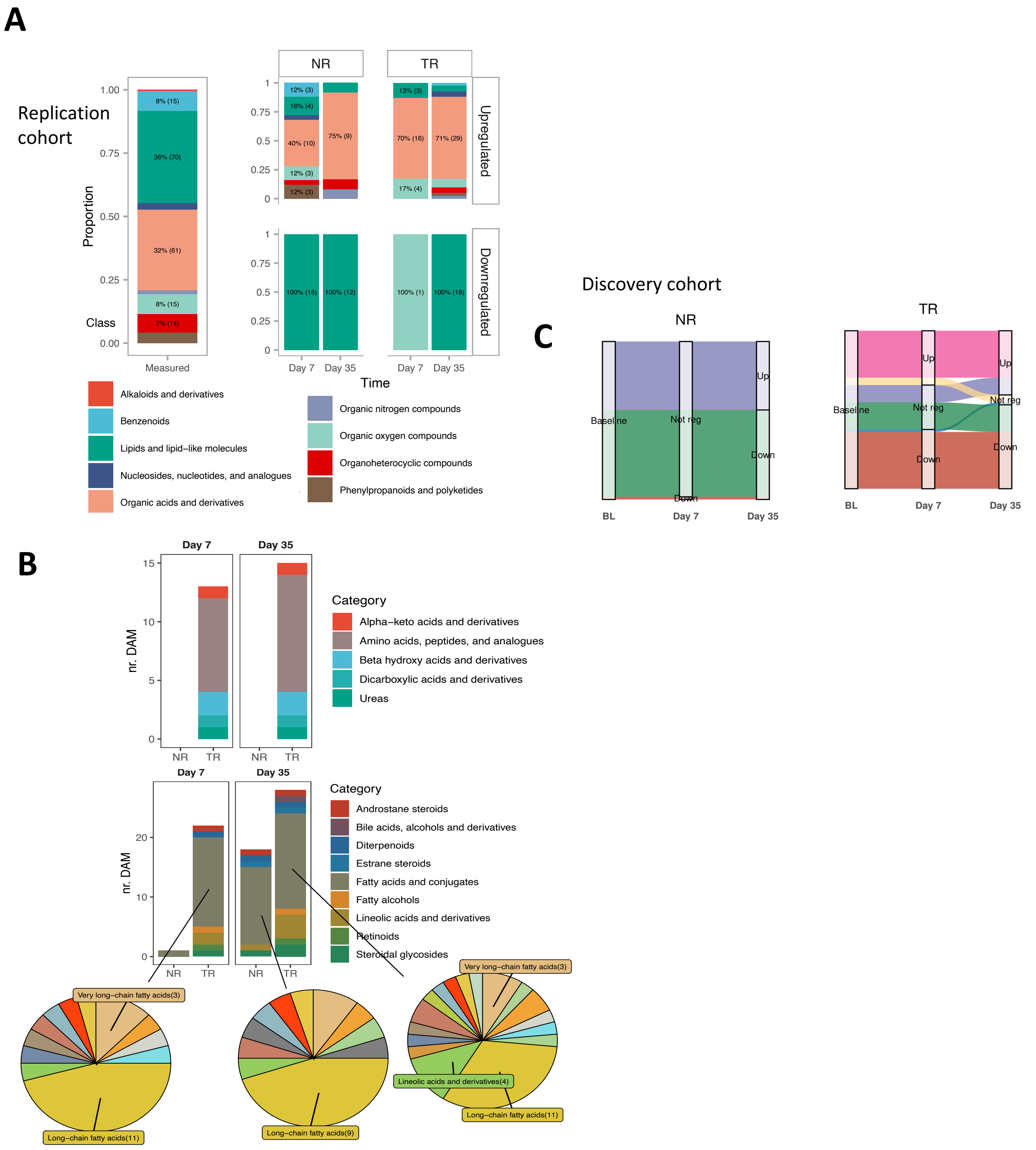

Figure S3. Metabolomic response to TIV (A) Annotation of the differentially abundant metabolites found in the replication cohort. “Measured” represents the metabolite proportions measured from different classes, TR and NR show significantly differentially abundant metabolites (nominal $p < 0.05$) at T3 and T4, respectively.**(B)** Subclass annotation of upregulated organic acids (top) and lipids and lipid-like molecules (bottom). These metabolites were significantly differentially abundant (adjusted $p < 0.05$) in the discovery cohort. Pie charts indicate the distribution of significant metabolites within the fatty acids and conjugates classification. **(C)** Alluvial plot showing the shared or unique metabolites differentially expressed for TRs (left) and NRs (right) at the different time points compared to pre-vaccination in the discovery cohort (n=81).

Figure S3

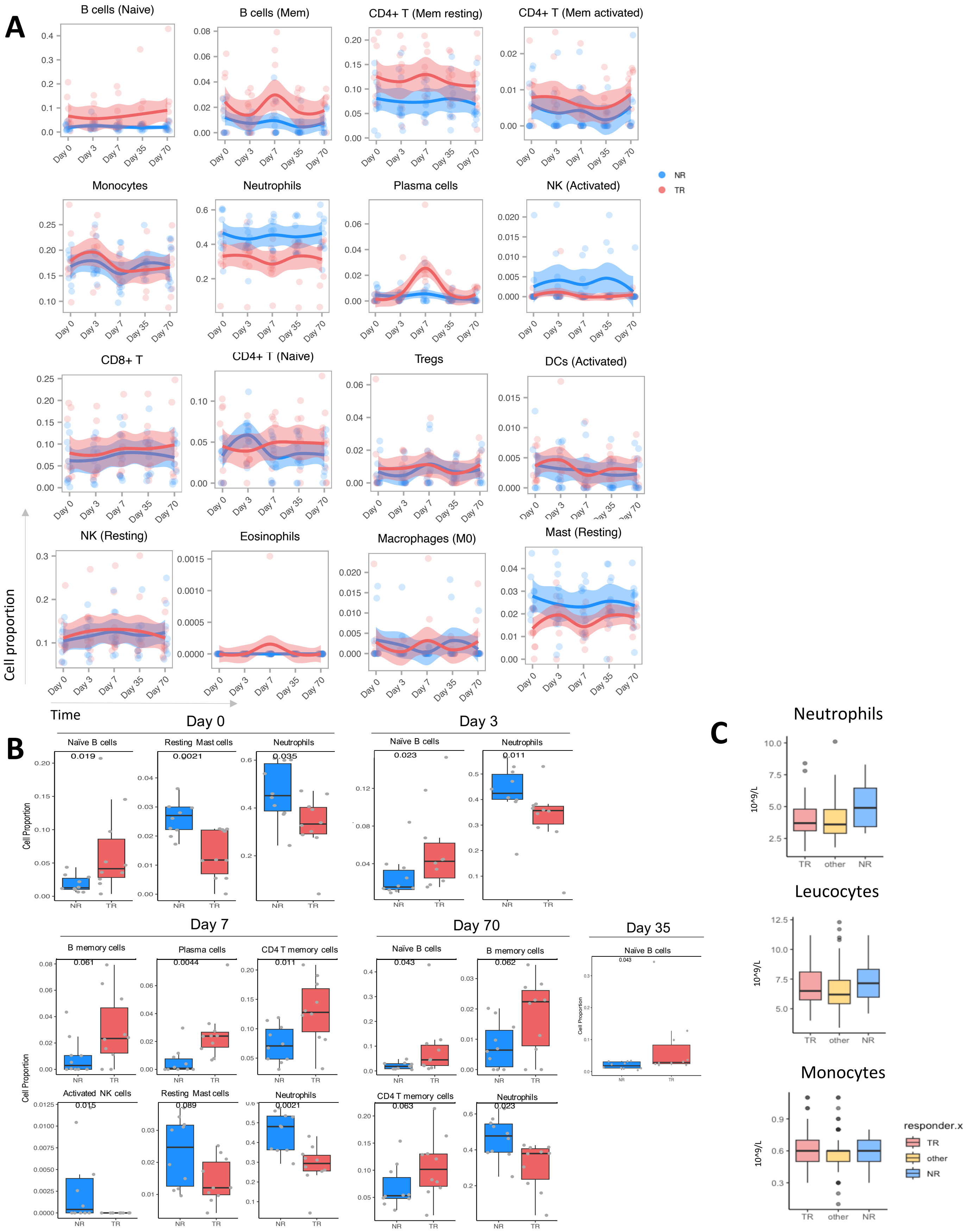

Figure S4. Differences in cellular populations between responders and non-responders (A) Deconvoluted cellular populations were estimated using CIBERSORT from bulk transcriptomics data (n=10 TR, n=10 NR) across five different timepoints. Each point indicates a sample (NR=blue, TR-red). The curve is a smooth fitted line with standard error of the estimation.**(B)** Cell Types showing significant differences in estimated cell proportions as in A between TRs and NRs at each time point. **(C)** CBC at T1 (pre-vaccination) for Neutrophils, Leucocytes and Monocytes (n = 200 donors). Samples are stratified by their responder status: TR, Other or NR.

Figure S4

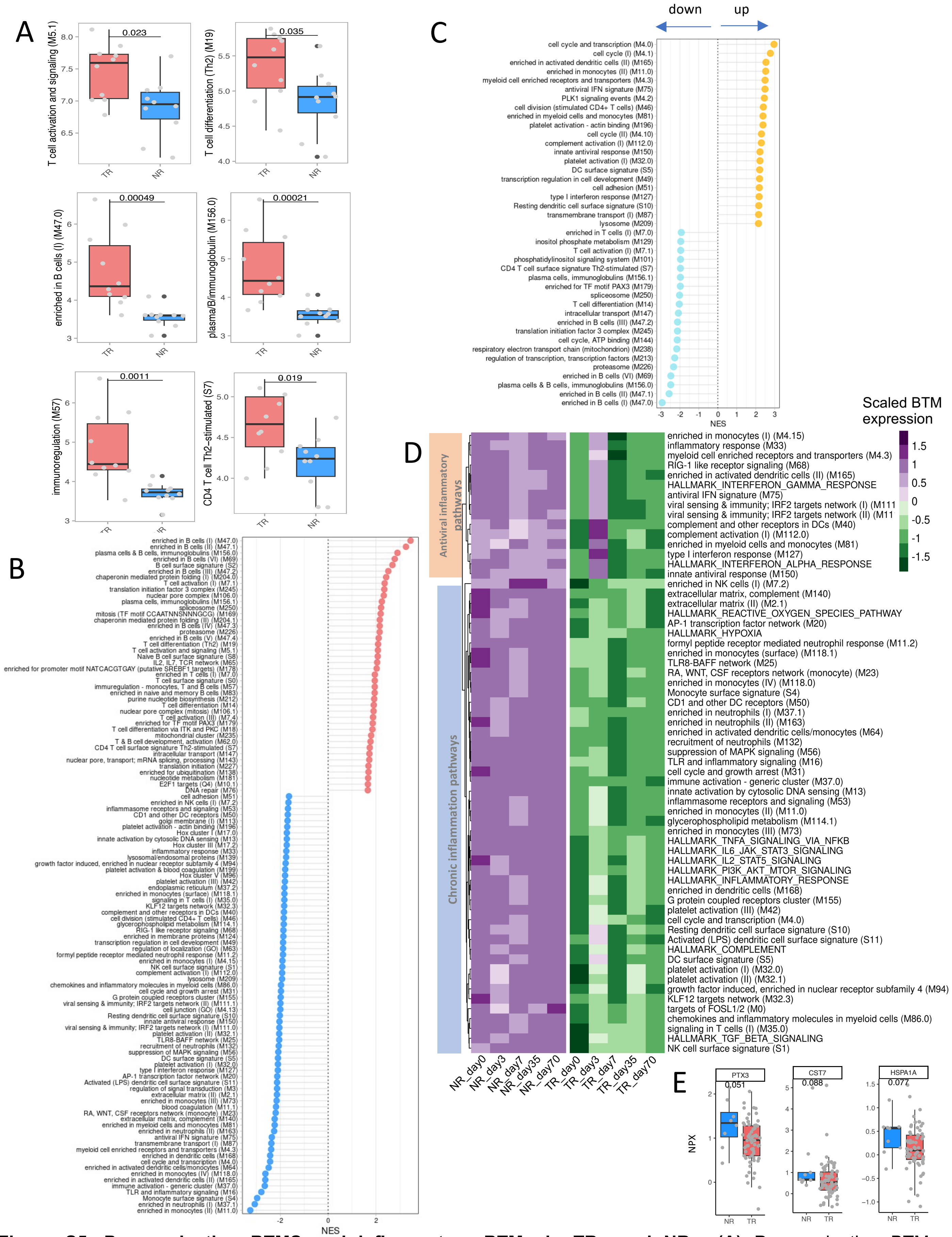

Figure S5. Pre-vaccination BTMS and inflammatory BTMs in TRs and NRs. (A) Pre-vaccination BTMs significantly discriminating the highest and lowest elderly responders. Each dot refers to a donor, p value generated using Wilcoxon Ranksum test (**B)** All significant BTMs upregulated in TRs and NRs at pre-vaccination stage.**(C)** Top 20 significant BTMs upregulated and downregulated in TRs 3 days post vaccination. **(D)** Chronic inflammatory pathways upregulated in NRs and antiviral pathways transiently upregulated in TRs. **(E)** Boxplot of pre-vaccination abundance of proteins enriched in chronic inflammatory pathways in NRs and TRs.

Figure S5

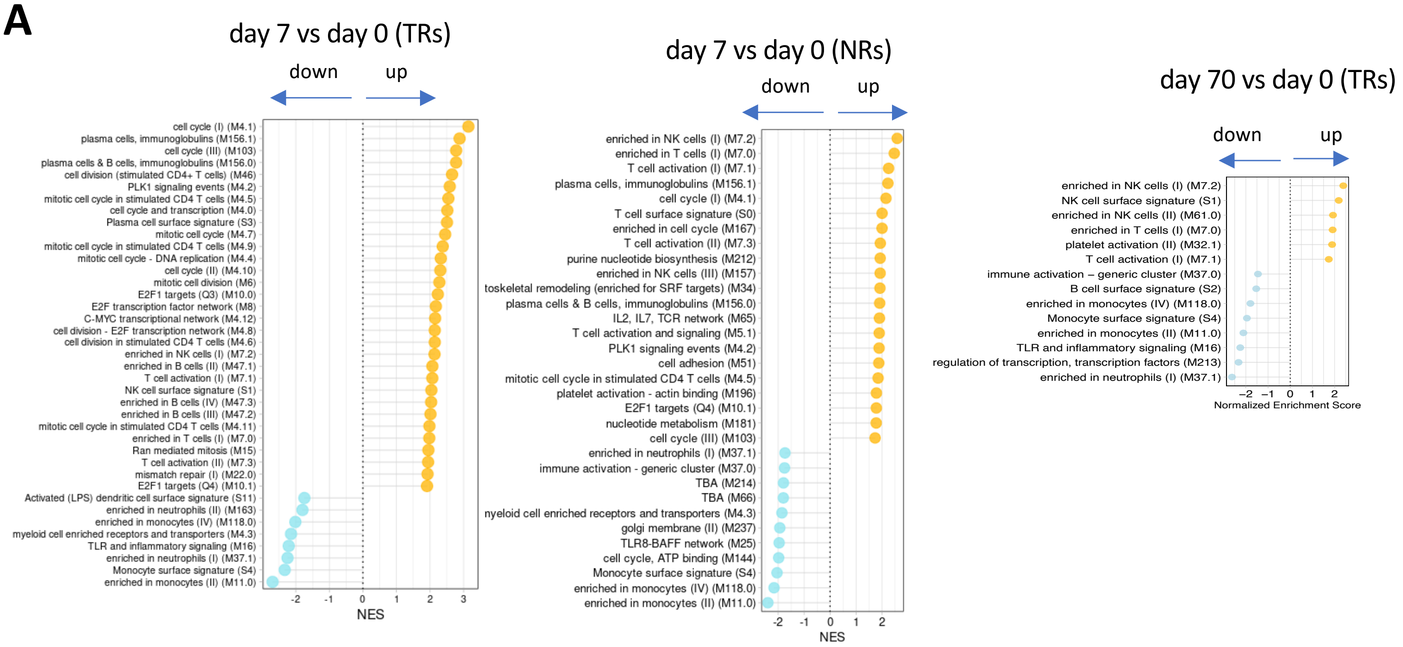

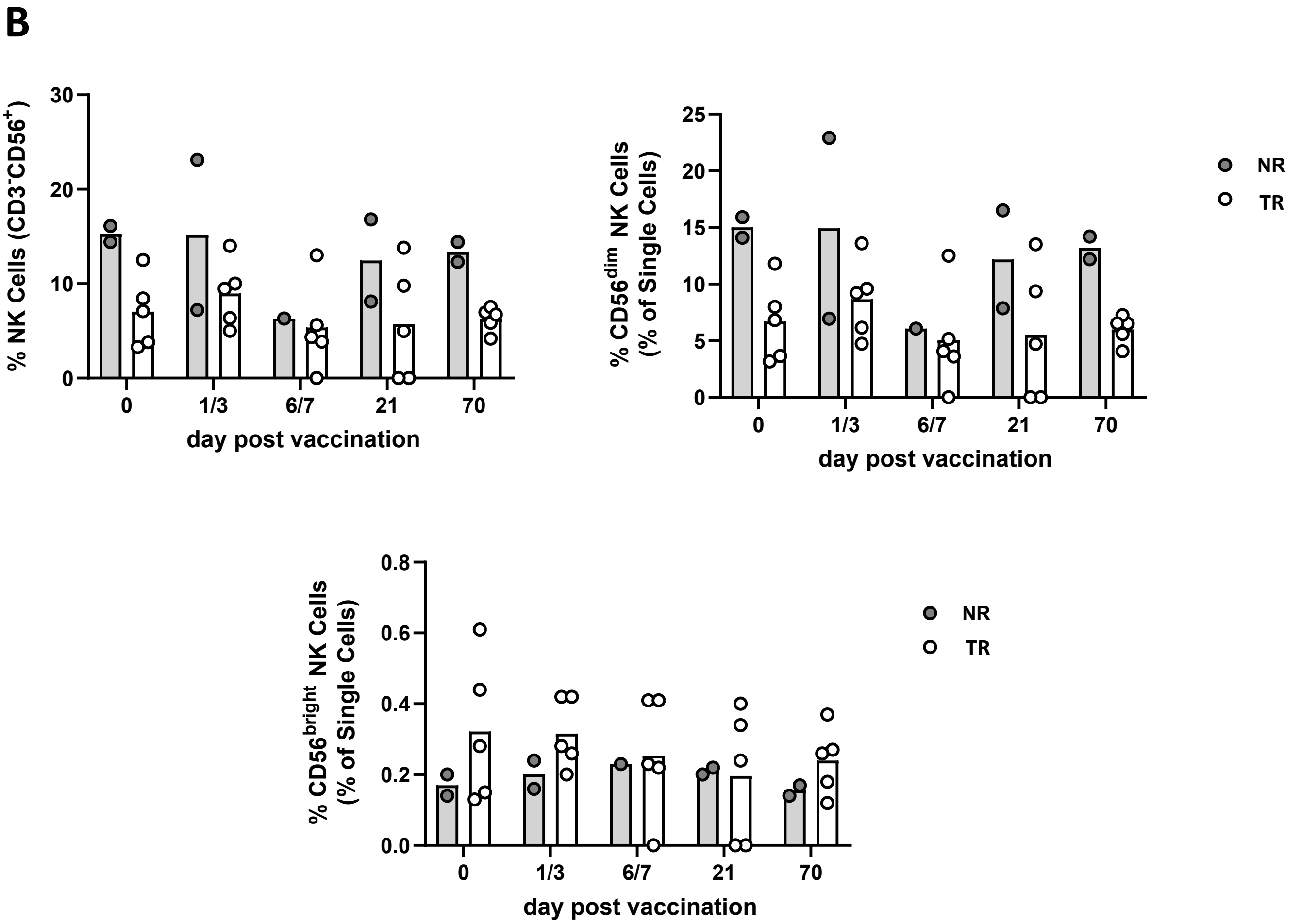

Figure S6. BTMs at day 7 and day 70 post-vaccination from discovery cohort and NK cell flow cytometry results from the replication cohort. (A) Significant BTMs in TRs and NRs when comparing day 7 vs day 0 (left and middle) and day 70 vs day 0 in TRs (right) **(B)** Flow cytometry results of Total NK cell proportions (top), CD56dim (middle) and CD56bright (bottom) from NRs (n=2, except day 6/7 where n=1) and TRs (n=5).

Figure S6

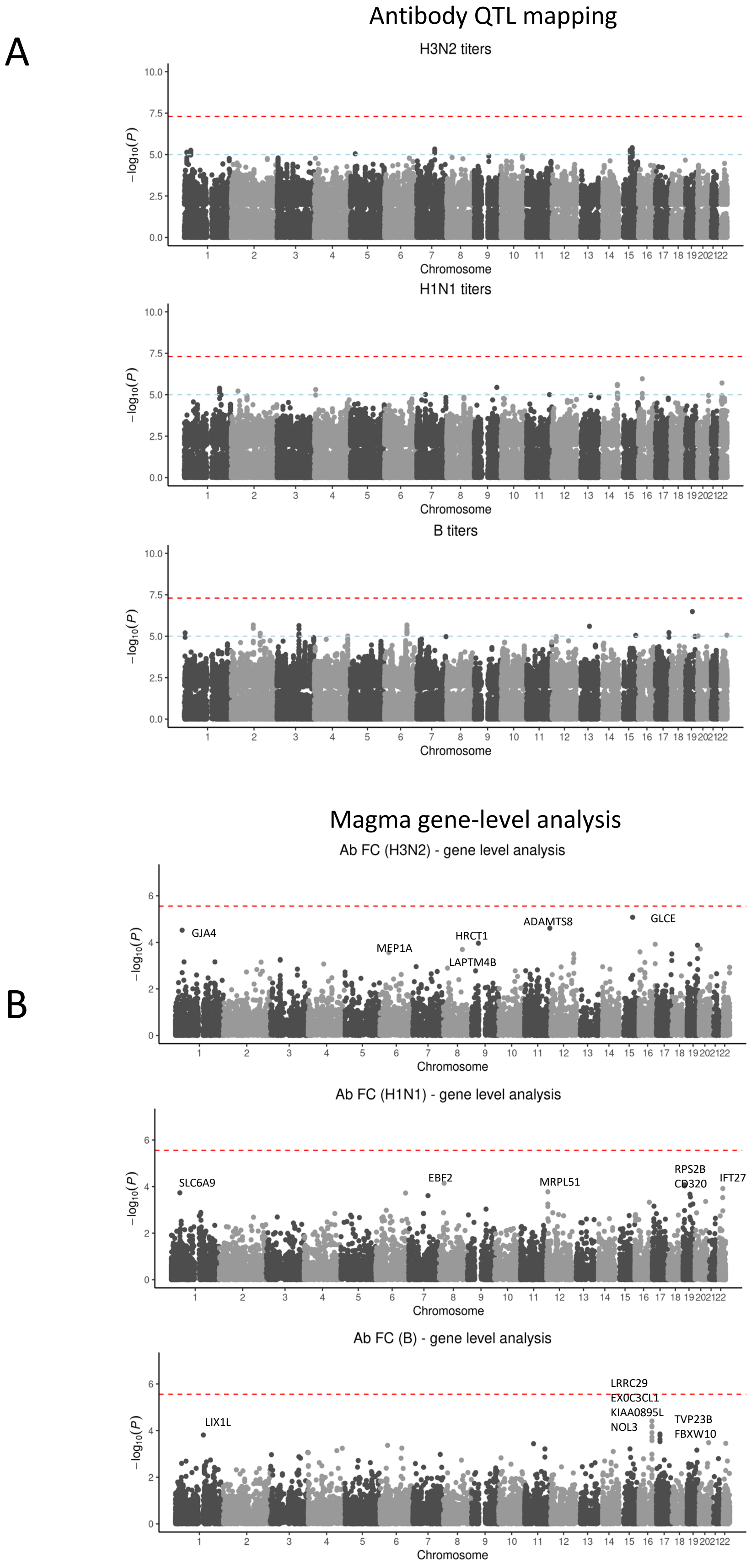

Figure S7. **Antibody quantitative trait loci mapping**. **(A)** Manhattan plot showing variant-level p-values. We mapped the serological response to vaccination (that is, antibody fold-change for each of three influenza strains upon vaccination). Genome-wide significance was set $5x10^{-8}$ (red line) **(B)** Gene-level p-values of antibody fold-change quantitative trait loci mapping as aggregated by MAGMA. Significance was determined by the number of genes tested: 18651 genes / 0.05 (red line).

Figure S7

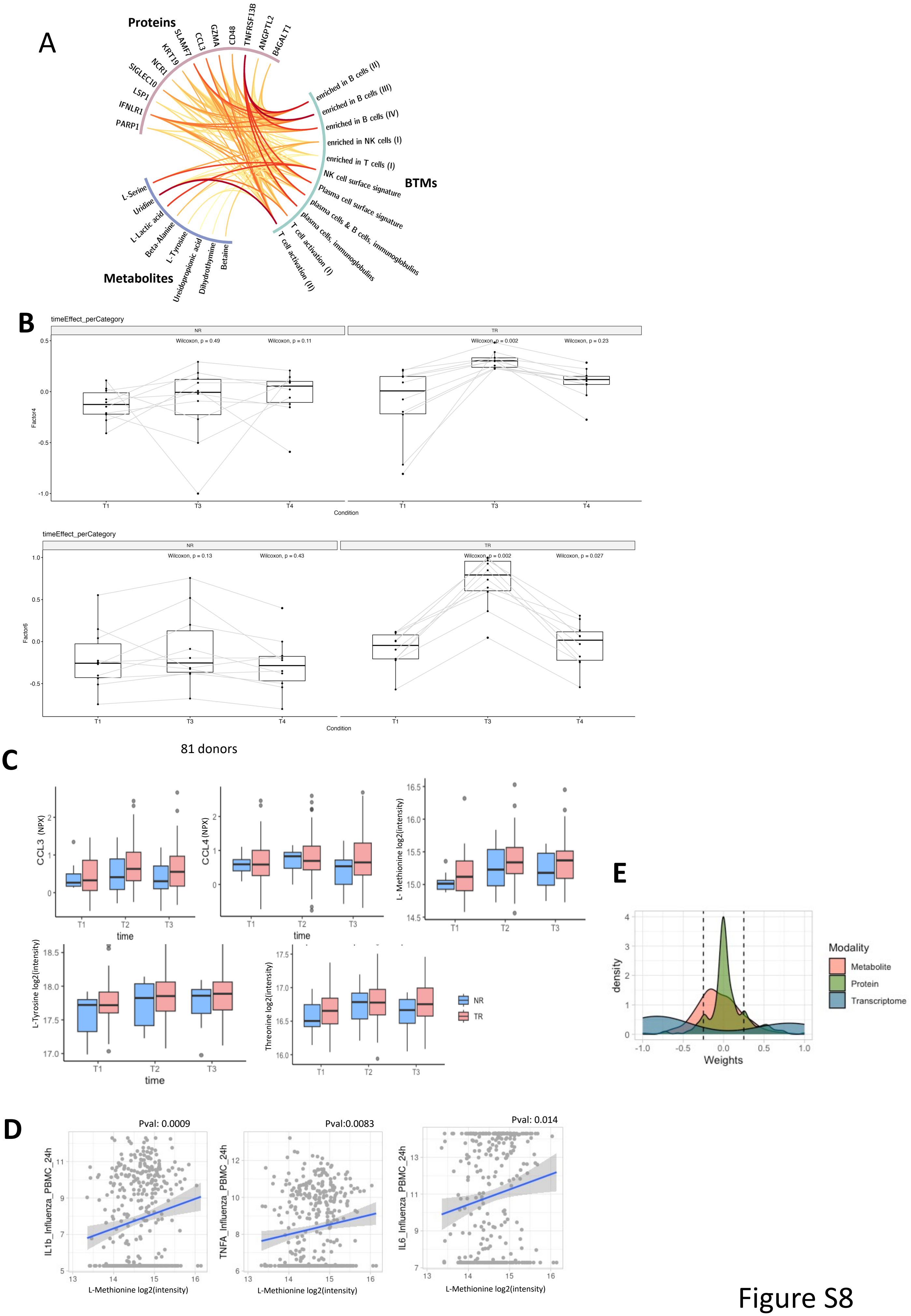

A
Proteins
SLAMF7
KRT19
CCL3
GZMA
CD48
TNFRSF13B
ANGPTL2
B4GALT1
NCR1
SIGLEC10
LSP1
IFNLR1
PARP1
enriched in B cells (II)
enriched in B cells (III)
enriched in B cells (IV)
enriched in NK cells (I)
enriched in T cells (I)
NK cell surface signature
Plasma cell surface signature
plasma cells & B cells, immunoglobulins
plasma cells, immunoglobulins
T cell activation (I)
T cell activation (II)
BTMs
L-Serine
Uridine
L-Lactic acid
Beta-Alanine
L-Tyrosine
Ureidopropionic acid
Dihydrothymine
Betaine
Metabolites
B
timeEffect_perCategory
NR
TR
Wilcoxon, p = 0.49
Wilcoxon, p = 0.11
Wilcoxon, p = 0.002
Wilcoxon, p = 0.23
Factor4
Condition
timeEffect_perCategory
NR
TR
Wilcoxon, p = 0.13
Wilcoxon, p = 0.43
Wilcoxon, p = 0.002
Wilcoxon, p = 0.027
Factor6
Condition
C
81 donors
CCL3 (NPX)
CCL4 (NPX)
L- Methionine log2(intensity)
L-Tyrosine log2(intensity)
Threonine log2(intensity)
time
NR
TR
E
density
Weights
Modality
Metabolite
Protein
Transcriptome
D
Pval: 0.0009
Pval:0.0083
Pval: 0.014
IL1b_Influenza_PBMC_24h
TNFA_Influenza_PBMC_24h
IL6_Influenza_PBMC_24h
L-Methionine log2(intensity)

Figure S8

Figure S8. Multi-omics integration capturing different dynamics in TRs and NRs. (A) Integration of proteins and metabolites with increased abundance at 7 days post-vaccination with transcriptome pathways in TRs. Linear mixed models were used to estimate the association between protein or metabolite abundance and mean transcriptomic pathway activity (Methods). Drawn links are significant at padj < 0.05. Color and width for the links are based on p-values, where wide red links indicate increased significance. **(B)** Factor 4 and Factor 6 showing time variation captured in TRs. Factor 4 is exclusively explained by transcriptome while factor 6 is explained by both transcriptome and proteome. **(C)** Abundance of factor 3 top proteins and metabolites in all 81 TRs and NRs. **(D)** Methionine positive correlation with cytokine production post influenza stimulation in independent 500 healthy individuals cohort. **(E)** Distribution of scaled weights attributed to proteins, metabolites and enrichment score for transcripts. Molecules with scaled weights of absolute value > = 0.25 were considered for network generation in Figure 4E.

Figure S8

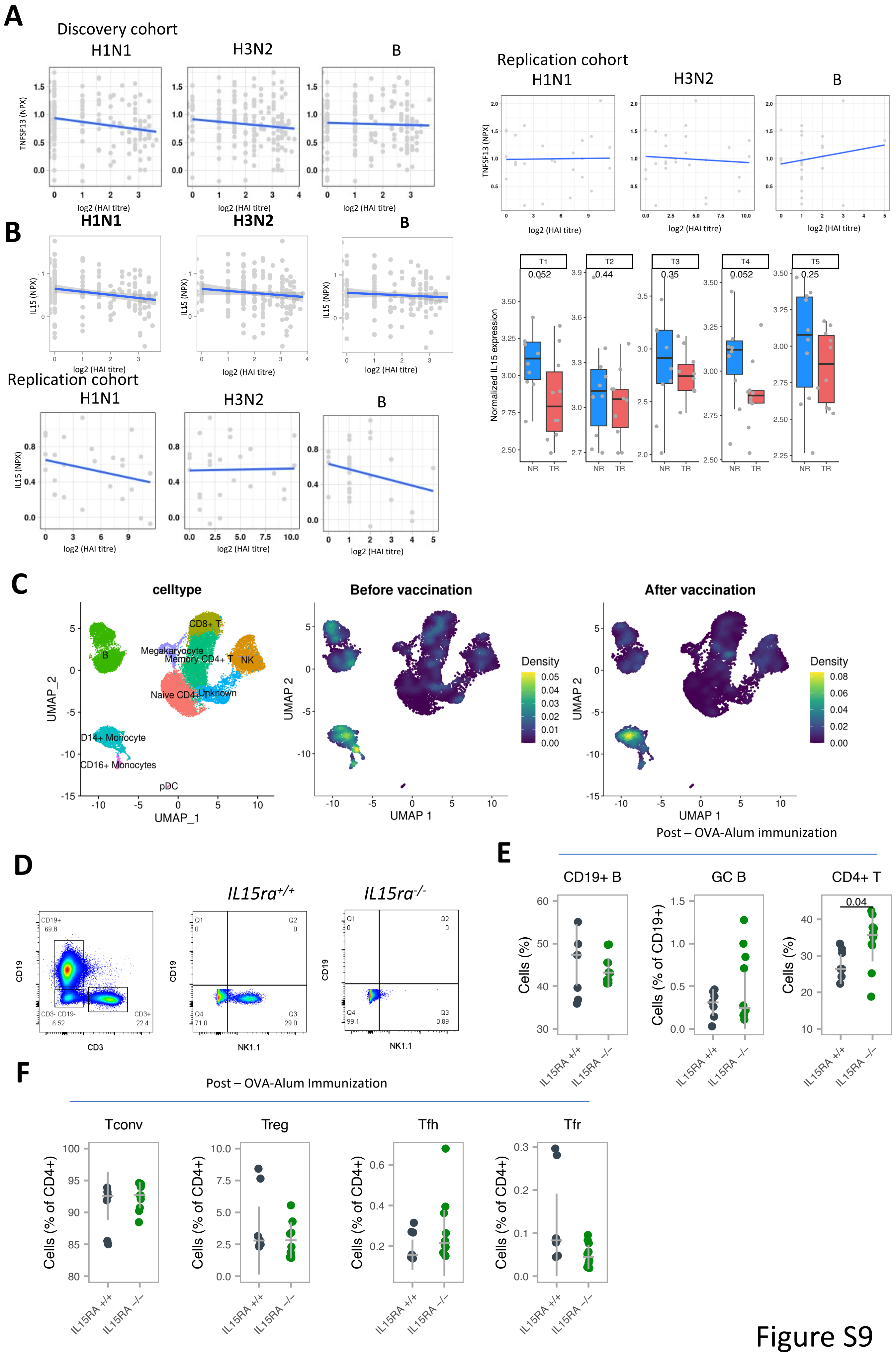

A
Discovery cohort
H1N1
H3N2
B
TNFSF13 (NPX)
log2 (HAI titre)
Replication cohort
H1N1
H3N2
B
TNFSF13 (NPX)
log2 (HAI titre)
B
H1N1
H3N2
B
IL15 (NPX)
log2 (HAI titre)
Replication cohort
H1N1
H3N2
B
IL15 (NPX)
log2 (HAI titre)
T1
0.052
T2
0.44
T3
0.35
T4
0.052
T5
0.25
Normalized IL15 expression
NR
TR
C
celltype
Before vaccination
After vaccination
CD8+ T
Megakaryocyte
Memory CD4+ T
NK
B
Naive CD4+
Unknown
D14+ Monocyte
CD16+ Monocytes
pDC
UMAP_1
UMAP_2
UMAP 1
UMAP 2
Density
Post – OVA-Alum immunization
D
IL15ra+/+
IL15ra-/-
CD19+
69.8
CD3- CD19-
6.52
CD3+
22.4
CD19
CD3
Q1
0
Q2
0
Q4
71.0
Q3
29.0
Q4
99.1
Q3
0.89
NK1.1
E
CD19+ B
GC B
CD4+ T
0.04
Cells (%)
Cells (% of CD19+)
IL15RA +/+
IL15RA –/–
F
Post – OVA-Alum Immunization
Tconv
Treg
Tfh
Tfr
Cells (% of CD4+)
IL15RA +/+
IL15RA –/–

Figure S9

Figure S9. Pre-vaccination correlates and *IL15RA-/-* mice experiments (**A)** Plot of TNFSF13/APRIL against log2 antibody fold change of each strain in discovery (left) and replication cohort (right). **(B)** Plot of IL15 against antibody fold change of each strain in discovery (top) and replication cohort (bottom). Boxplot of *IL15* transcriptome expression in TRs and NRs. **(C)** Expression of IL-15 in human PBMCs using single-cell RNA sequencing data.**(D)** Representative flow cytometry plots showing the frequency of B cells (CD19+) and T cells (CD3+) in the spleen of IL15RA$^{-/-}$ mice (left plot), and the NK cell frequency (NK1.1+) among splenocytes from IL15RA$^{+/+}$ and IL15RA$^{-/-}$ mice (two plots on the right). **(E)** The dot plots represents post-immunization with OVA-Alum frequency of total B cells, GC B cells, total CD4+T cells and **(F)** CD4+Foxp3- Tconv cells, CD4+Foxp3+ Treg cells, Tfh, and Tfr cells in inguinal lymph nodes from IL15RA+/+ and IL15RA-/- mice. Pooled data from two independent experiments; n=8 to 10.

Figure S9

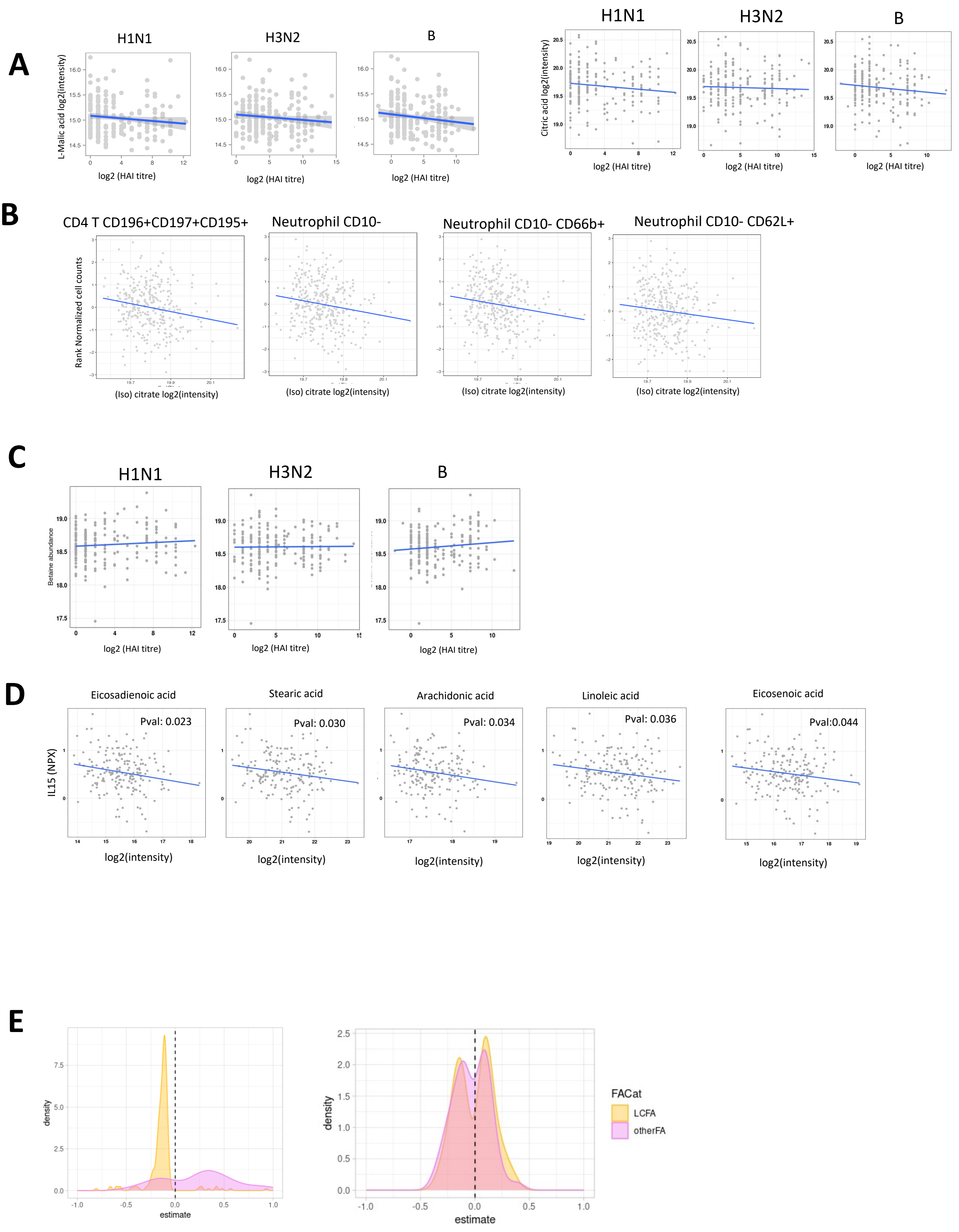

Figure S10. Pre-vaccination metabolite correlates with antibody fold change. (A) Plot of malic acid and citric acid against antibody fold change for each strain. **(B)** Plot of citric acid abundance against cell proportions calculated from 300BCG cohort. **(C)** Plot of betaine(top) abundance against antibody fold change for each strain. **(D)** Additional unsaturated long chain fatty acids negatively correlated to IL15. **(E)** Estimate of protein (42) associations to all fatty acids for ~200 elderly donors (left) and to ~ 500 young donors (right). In the elderly, LCFA negatively correlated to most proteins while other fatty acids (FAs) showed both positive and negative correlation while no such pattern was observed in the younger cohort. Associations with p-value <0.05 plotted.

Figure S10

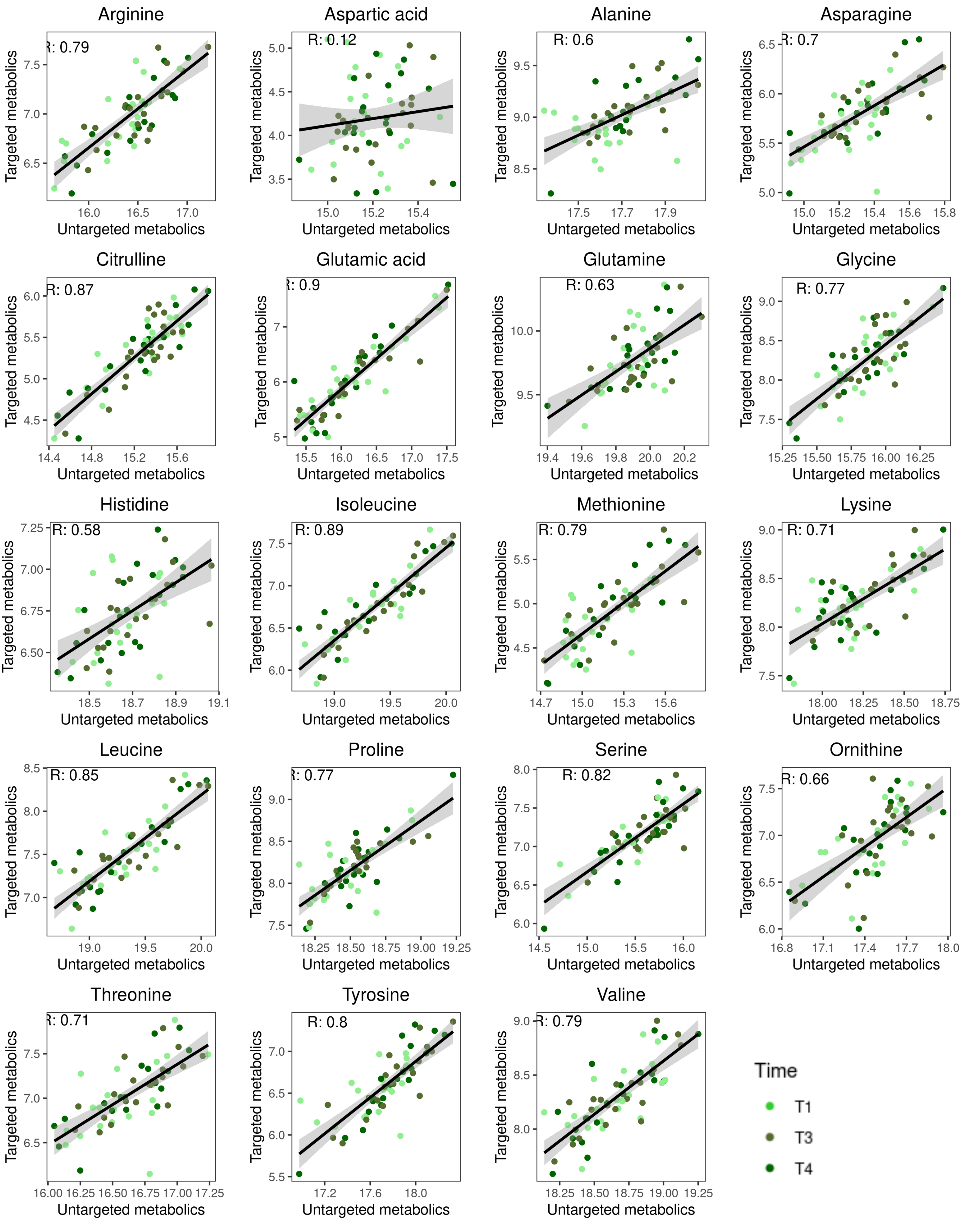

Figure S11 Replicability of untargeted metabolomic profiles. Correlation of 19 primary amino acids detected in both, the untargeted and targeted metabolomics dataset. Each dot indicates a sample, coloured for time (10TRs, 10NRs across three timepoints). We observe good replicability for the primary amino acids.

Figure S11

Tables ST1 to ST8

Supplementary Table 1. Differentially abundant proteins in HRs and LRs of each strain post-vaccination.

Supplementary Table 2. Differentially abundant metabolites in HRs and LRs of each strain post-vaccination.

Supplementary Table 3. Differentially abundant proteins in TRs and NRs post-vaccination.

Supplementary Table 4. Differentially abundant metabolites in TRs and NRs post-vaccination.

Supplementary Table 5. Metabolome over-representation results for TRs at day 7 vs day 0

Supplementary Table 6. GSEA using BTMs comparing TRs to NRs at all time points.

Supplementary Table 7. GSEA using BTMs comparing TRs to NRs at only pre-vaccination time point.

Supplementary Table 8. GSEA using BTMs comparing day 3 vs day 0 in TRs.
